# Structure of the R2 non-LTR retrotransposon initiating target-primed reverse transcription

**DOI:** 10.1126/science.adg7883

**Published:** 2023-04-06

**Authors:** Max E. Wilkinson, Chris J. Frangieh, Rhiannon K. Macrae, Feng Zhang

**Affiliations:** 1 Howard Hughes Medical Institute; Cambridge, MA 02139, USA.; 2 Broad Institute of MIT and Harvard; Cambridge, MA 02142, USA.; 3 McGovern Institute for Brain Research, Massachusetts Institute of Technology; Cambridge, MA 02139, USA.; 4 Department of Brain and Cognitive Science, Massachusetts Institute of Technology; Cambridge, MA 02139, USA.; 5 Department of Biological Engineering, Massachusetts Institute of Technology; Cambridge, MA 02139, USA.; 6 Department of Electrical Engineering and Computer Science, Massachusetts Institute of Technology; Cambridge, MA 02139, USA.

## Abstract

Non-LTR retrotransposons, or Long Interspersed Nuclear Elements (LINEs), are an abundant class of eukaryotic transposons that insert into genomes by target-primed reverse transcription (TPRT). During TPRT, a target DNA sequence is nicked and primes reverse transcription of the retrotransposon RNA. Here, we report the cryo-electron microscopy structure of the *Bombyx mori* R2 non-LTR retrotransposon initiating TPRT at its ribosomal DNA target. The target DNA sequence is unwound at the insertion site and recognized by an upstream motif. An extension of the reverse transcriptase (RT) domain recognizes the retrotransposon RNA and guides the 3′ end into the RT active site to template reverse transcription. We used Cas9 to retarget R2 *in vitro* to non-native sequences, suggesting future use as a reprogrammable RNA-based gene-insertion tool.

## One-Sentence Summary

A retrotransposon structure shows the principles of target DNA selection and self RNA recognition.

**Fig. 1.**
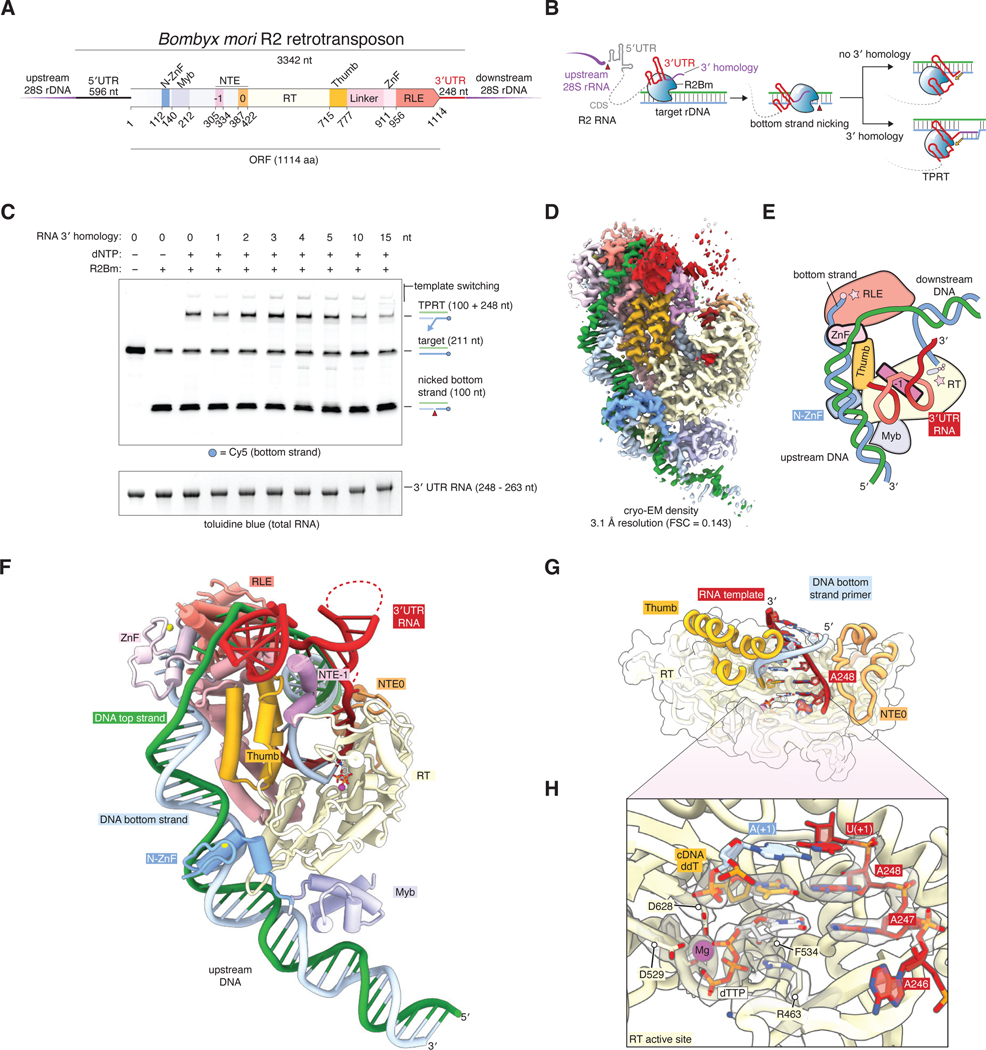
Cryo-EM structure of the R2Bm retrotransposon. (**A**) Domains of the R2Bm retrotransposon. ZnF, zinc finger; NTE, N-terminal extension; RT, reverse transcriptase; RLE, restriction-like endonuclease. (**B**) Schematic of target-primed reverse transcription (TPRT). (**C**) Denaturing gel of in vitro TPRT reactions on a labeled 211-bp 28S DNA target. The same gel was visualized by Cy5 fluorescence and toluidine blue staining. (**D**) Cryo-EM density of the R2Bm TPRT complex. (**E**) Cartoon of the cryo-EM structure. Stars represent active sites. (**F**) Atomic model for the R2Bm TPRT complex. (**G**) Reverse transcriptase domain and template/primer duplex. (**H**) Reverse transcriptase active site. Cryo-EM density is shown as a gray transparent surface.

Non-long terminal repeat (non-LTR) retrotransposons are the most abundant class of mobile genetic element (MGE) in the human genome, mostly represented by the LINE-1 and SINE (or Alu) elements (1). Despite their prevalence and contribution to genetic diversity and dysregulation through mutagenicity and recombination (1–3) and their prospective use as gene insertion tools, there is much left to understand about their mobility mechanisms (4). Pioneering research on the *Bombyx mori* (silk moth) R2 element (R2Bm), which selectively inserts into the 28S rRNA gene, has contributed significantly to our understanding of this type of MGE (5). R2, like all non-LTR retrotransposons, encodes an open reading frame (ORF) with DNA-binding, endonuclease, and reverse transcriptase activities (Fig. 1A). The endonuclease domain (restriction-like endonuclease, RLE) nicks the target DNA, and the reverse transcriptase domain uses the exposed 3’ end from the nick to prime reverse transcription of the R2 RNA, resulting in a new genomic copy of the R2 element (Fig. 1B) (6, 7). This process is called target-primed reverse transcription (TPRT), and is characteristic of non-LTR retrotransposons and their group II intron ancestors (8, 9). The nicked strand that primes reverse transcription is referred to as the bottom strand. Complementarity between the bottom strand and the 3′ end of the R2 RNA (3′ homology) is not required to initiate reverse transcription (10) Non-LTR retrotransposons are specific for reverse transcribing their own RNA; for R2, this specificity requires an element in the 3′UTR but the precise motif has not been located (11). It is also unclear how R2 specifically recognizes the 28S rRNA target gene, or how DNA nicking is coupled to reverse transcription within the same protein. To address these questions, we solved a cryo-EM structure of the *Bombyx mori* R2 protein (R2Bm) initiating TPRT at the 28S rRNA gene using its own 3′UTR. The structure reveals an extensive interface with the target DNA, a small core region of the 3′UTR required for TPRT, and shows that R2Bm can be engineered to reprogram its insertion site.

### Reconstitution and cryo-EM structure of an R2 TPRT complex

We overexpressed R2Bm in *Escherichia coli* and purified it to apparent homogeneity (fig. S1). The purified protein was active in vitro, reproducing previously found biochemical activities, including RNA-stimulated nicking of the target DNA bottom strand, site-specific TPRT when supplied with in vitro transcribed 3′UTR RNA, and low levels of template jumping (Fig. 1C) (6, 12). It is unclear if 3′ homology is required for TPRT in vivo; however, consistent with previous findings, we found that downstream sequences up to 10 nt do not inhibit activity in vitro (Fig. 1C) (10). Sequencing of TPRT junctions confirmed that homology-mediated TPRT is more likely to initiate reverse transcription at the 3′ end of the 3′ UTR rather than skipping bases or inserting untemplated nucleotides (fig. S2). (10). To assemble a complex stalled during initiation of TPRT, we incubated R2Bm with target DNA, 3′UTR RNA, and the chain-terminator nucleotide 2′,3′-dideoxythymidine (ddT), which mimics the first nucleotide incorporated in the TPRT reaction (dT) but does not allow further elongation. Purified TPRT complexes contained stoichiometric amounts of R2Bm, 3′UTR RNA, and target DNA with > 99% of the bottom strand nicked (fig. S1). Initial attempts at cryo-EM imaging failed due to the preferred orientation and flexibility of the complex. To overcome these issues, we used a carbon support on the cryo-EM grid and added 5 nt of downstream 28S RNA sequence to the 3′ end of the 3′UTR RNA to stabilize the complex by forming a primer-template duplex with the target DNA bottom strand. With these modifications, we obtained a cryo-EM reconstruction of the R2 TPRT complex at 3.1 Å resolution (Fig. 1D, fig. S3–4, table S1).

The core of the R2Bm protein is a reverse-transcriptase domain (RT) similar to group II intron RTs (13), followed by a C-terminal ɑ-helical thumb domain and preceded by a characteristic N-terminal extension domain (NTE0) implicated in template switching (14), but the R2Bm RT includes a further N-terminal extension (NTE-1) that binds the 3′UTR RNA (Fig. 1E, F) (15). Preceding the NTE-1 element are two DNA binding domains: the N-terminal C2H2 zinc finger domain (N-ZnF) and a Myb domain. C-terminal to the thumb domain lies an ɑ-helical linker domain that packs against the thumb, followed by a CCHC zinc-finger domain (ZnF) conserved in many LINE ORFs (4). The ZnF then links to the C-terminal RLE domain, which cleaves the target DNA. This domain arrangement closely resembles Prp8 (13, 16, 17), the core protein of the spliceosome, underscoring the close relationship between pre-mRNA splicing and retrotransposons.

There are several key interactions between the R2Bm protein, 3′UTR RNA, and target DNA (Fig. 1E, F). The two strands of the target DNA separate around the ZnF domain, with the bottom strand feeding into the RLE active site where the scissile phosphate remains bound, while the top strand snakes along the opposing surface of the RLE. The RT active site contains a heteroduplex formed by the nicked bottom strand of the target DNA (5′ to the cleavage site) and the 5 nt of 28S RNA homology extension beyond the 3′UTR RNA (Fig. 1G). This target heteroduplex is surrounded by residues important for RT activity (18), and the cryo-EM density shows incorporation of the ddT chain terminator nucleotide into the bottom strand (Fig. 1H). The 5′ end of the bottom strand remains base-paired to the top strand as it leaves the RLE, and this downstream DNA region has weak cryo-EM density, suggesting it is not tightly bound by R2Bm. The 248-nt 3′UTR RNA is mostly not resolved in the cryo-EM density except for a core 40-nt region, which wraps around the NTE-1 ɑ helix of R2Bm and the 3′ end of which is guided into the RT active site via the NTE0 domain.

### R2Bm recognizes a sequence motif upstream of the cleavage site

**Fig. 2.**
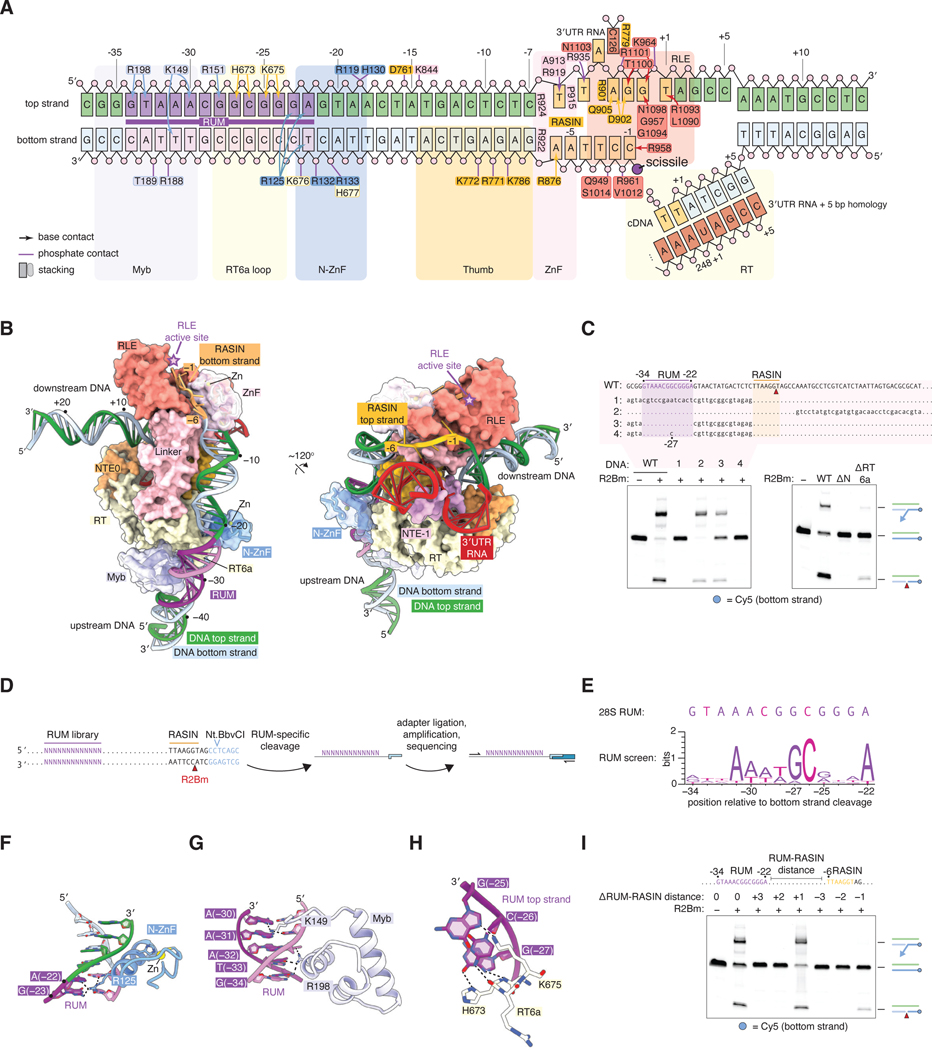
Target DNA recognition upstream of the R2 cleavage site. (**A**) Schematic of interactions with the target DNA. Bases are numbered relative to the bottom strand cleavage site. Positions of protein domains are shown by shaded rectangles. (**B**) Structure of R2Bm around the upstream DNA sequences. (**C**) Effect of upstream DNA mutations on target cleavage. The schematic shows the sequences of five DNA sequences tested in top-strand sense; dots represent bases identical to wildtype. Red triangle, bottom strand cleavage site. Denaturing gels show in vitro TPRT reactions on labeled 211-bp 28S DNA targets. ΔN, deletion of N-terminal N-ZnF and Myb domains. ΔRT6a, deletion of residues 672 – 677 (DGHRKK) of the RT6a loop. (**D**) Screen for identifying active RUM sequences. Nicking sites of R2Bm and the restriction endonuclease Nt.BbvCI are shown by triangles. (**E**) Sequence logo for sequences enriched in the RUM screen. (**F, G, H**) Details of interactions between the target DNA and the N-ZnF, Myb, and RT6a loop. (**I**) Effect of altering the distance between the RUM and RASIN motifs. Denaturing gel shows in vitro TPRT reactions on labeled 211-bp 28S DNA targets.

The target 28S DNA sequence has extensive interactions with R2Bm (summarized in Fig. 2A). Upstream bases from –38 to –7 and downstream bases from +6 to +21 are respectively paired, whereas the 11 base pairs from –6 to +5 are melted around the RLE domain (bases are numbered relative to the bottom strand cleavage site). The upstream DNA has a 40° bend and binds along the surface of the RT, linker, and thumb domains in a manner similar to the DNA in a recent group IIC intron maturase structure (Fig. 2B, fig. S5, S6) (19). Many of the contacts between R2Bm and the DNA are via the phosphate backbone, suggesting that they are not sequence-specific. Based on the structure, however, we predicted that two regions are key for sequence-specific DNA recognition by R2Bm: a 13-bp upstream motif from –34 to –22, which is bound by the N-terminal N-ZnF and Myb domains, and the 7 bp from –6 to +1, which are bound by the RLE (Fig. 2A). We term these regions the Retrotransposon Upstream Motif (RUM) and Retrotransposon-Associated INsertion site (RASIN), respectively.

Consistent with the importance of the RUM region for R2 activity, mutating the entire upstream sequence between –38 to –7 eliminated bottom strand cleavage, whereas mutating the downstream sequences between +6 and + 37 preserved wild-type levels of bottom strand cleavage and TPRT (Fig. 2C) (20). Adding just the 13-bp RUM region to the upstream mutant at positions –34 to –22 restored near-wild-type activity, whereas a point mutant RUM (G_–27_ to C) did not rescue activity (Fig. 2C). This region of the target was strongly protected in a previous DNase footprinting assay (21). To systematically determine the importance of each base within the RUM, we performed an R2 cleavage assay on a DNA target with the upstream region (–38 to –7) mutated and the RUM (–34 to –22) replaced with a 13N library (Fig. 2D). Sequencing of cleaved targets revealed a consensus RUM sequence A_–31_WWWGCNNNA_–22_, where W is A/T and N is any nucleotide, with minor preferences in other positions (Fig. 2E). This consensus is a close match to the wild-type 28S sequence A_–31_ACGGCGGGA_–22_ , with the differences underlined.

The RUM is recognized by three domains: N-ZnF, Myb, and an R2-specific insertion ‘6a’ in the RT domain between motifs 6 and 7 (Fig. 2B, fig. S7). The N-ZnF has the classical C2H2 fold with a zinc ion coordinated between an ɑ-helix and a β-hairpin, but unusually the ɑ-helix binds in the widened minor groove of the DNA from bases –18 to –23 instead of the typical major groove (Fig. 2F, fig. S6) (22). The preference for A at base –22 in the RUM is likely due to N-ZnF Arg125, which hydrogen bonds with the minor-groove–facing side of the A–T base pair (Fig. 2F). The Myb domain forms a typical three-helix bundle, with the third helix bound in the major groove from bases –31 to –34 (22) while its linker to N-ZnF engages with base –30 (Fig. 2G). This is reminiscent of other Myb–DNA structures, including telomere-interacting protein Rap1 (23). The Myb domain recognizes the A at base –31 via hydrogen bonds with Lys149 (Fig. 2G). Although Arg198 contacts bases at positions –33 and –34, these contacts appear not to be sequence specific, as the RUM screen showed only weak sequence preferences in this region (Fig. 2E, G). Deletion of the N-ZnF and Myb domains together (ΔN mutant) completely inhibits target DNA nicking and subsequent TPRT (Fig. 2C) (20). The central GC of the RUM is recognized by His673 and Lys675 of the loop 6a of the RT domain (Fig. 2H). Structural predictions suggest that this loop is unique among non-LTR RT domains to R2 proteins (fig. S7). We found that deletion of the 6a loop inhibits target DNA nicking (Fig. 2C). Finally, we found that the distance between the RUM and the bottom strand cleavage site (the RASIN) is important: increasing the distance by one base was tolerated, but further increase or any decrease to the distance inhibited target cleavage (Fig. 2I).

### Target DNA interactions at the cleavage and integration site

**Fig. 3.**
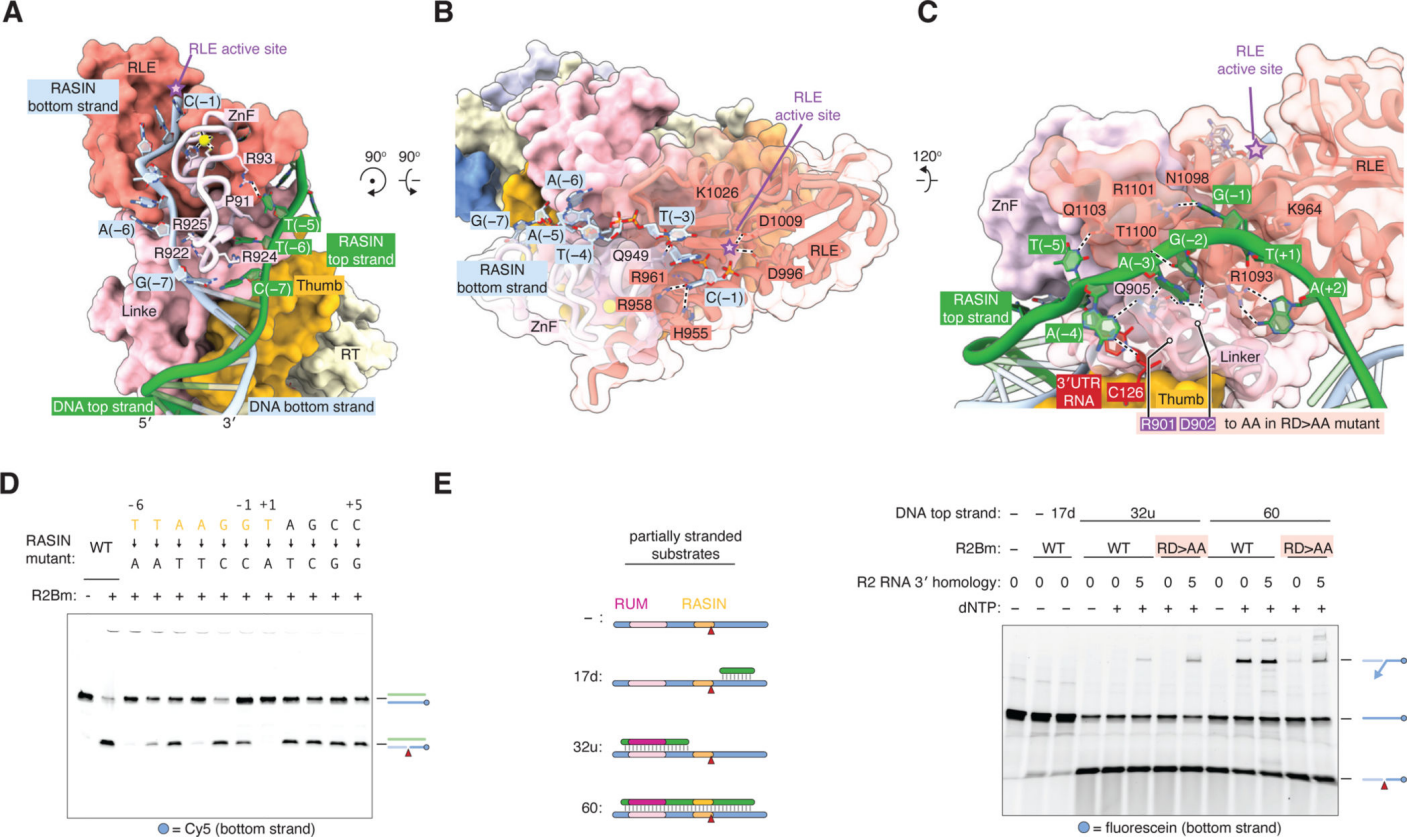
Target DNA recognition at the R2 cleavage site. (**A**) Interactions of the top and bottom strands of the target DNA with the ZnF domain of R2Bm. Star, RLE active site. (**B**) Interactions of the DNA bottom strand with the RLE domain. (**C**) Interactions of the DNA top strand with the RLE domain. Residues mutated in the RD>AA mutant are highlighted. (**D**) RASIN sequence requirements for bottom strand cleavage. The labeled 211-bp 28S DNA targets were incubated with R2Bm and 3′ UTR RNA in the absence of dNTPs. The reactions were analyzed with a denaturing gel. Mutations are notated in top-strand sense, but both strands were mutated. (**E**) Denaturing gel showing R2Bm cleavage and TPRT activity on partially-stranded substrates. Reactions contained a fluorescein-labeled 76-nt bottom strand. Reactions as indicated also contained 17 nt of downstream top strand sequence (17d), 32 nt of upstream top strand sequence (32u), or 60 nt of top strand sequence fully complementary to the bottom strand spanning the upstream and downstream regions. RD>AA; R2Bm R901A D902A.

The second key region for DNA target recognition by R2Bm is the target site for nicking by the RLE domain and R2 insertion, which we term the RASIN. In our structure, the 11 base pairs of the RASIN from –6 to +5 are melted around the RLE domain. The ZnF appears to act as the “zip,” stacking on the last upstream pair C–G(–7) with Arg922 and Arg924 and holding unzipped strands apart (Fig. 3A). Strand melting may be enhanced by the 40° bend in target DNA around the RUM (Fig. 1F). Bases –6 to –1 on the bottom strand then follow a cleft between the ZnF and the RLE, which adopts a canonical PD-(D/E)xK-family nuclease fold, but with the characteristic Lys1026 on an ɑ helix instead of the usual β strand (Fig. 3B) (24). This lysine, along with catalytic residues Asp996 and Asp1009, are 4 – 6 Å from the scissile phosphate of C(–1), suggesting C(–1) may be close to its position during catalysis of bottom strand cleavage. On the top strand, bases –6 to +2 all make extensive contacts along a cleft between the RLE and linker domains, except for A(–4), which flips out and contacts C126 of the 3′UTR (Fig. 3C). To determine the relative importance of the bases in the RASIN, we mutated each of the 11 bp individually and tested the effect on bottom strand cleavage. Mutating T(+1) to A abolished cleavage entirely, and mutating T(–6), T(–5), and A(–3) severely decreased activity, whereas other changes were tolerated (Fig. 3D). This suggests the following RASIN motif for cleavage, given in top strand sense: T_–6_TNANNT_+1_.

Because only the bottom strand of the RASIN enters the RLE active site, we tested the activity of R2Bm on a single-stranded DNA with the bottom strand sequence and found that it was cut, albeit weakly (Fig. 3E). Endonuclease activity was strongly stimulated by providing a 60-nt top strand spanning the RASIN and upstream and downstream sequences, but was similarly stimulated by a 32-nt top strand complementary only to the upstream region containing the RUM. A 17-nt top strand complementary to the downstream sequence did not stimulate activity (Fig. 3E). This suggests that the RUM in a double-stranded state is important for recruiting the R2Bm RLE to the RASIN bottom strand, and that the top strand of the RASIN, despite its extensive interaction with R2Bm, is dispensable for specific bottom strand cleavage. However, when we added deoxynucleotides to these reactions, TPRT activity was eliminated in the absence of the top strand from the RASIN downstream but was partially rescued if the 3′UTR RNA contained 3′ homology to the target site (Fig. 3E). The top strand RASIN bases A(–4), A(–3), and G(–2) are grasped by Arg901 and Asp902 of the R2Bm linker (Fig. 3C). We mutated these two residues to alanine and tested TPRT activity on a fully double-stranded substrate, and found that TPRT activity was reduced and partially rescued by 3′ homology (Fig. 3E). These results suggest two important factors for initiating TPRT when the 3′UTR RNA lacks 3′ homology. One: presence of a top strand downstream of the RASIN, which may help retain the nicked bottom strand, and two: contacts between R2Bm and the top strand RASIN, which help the nicked bottom strand “pivot” into the RT active site.

### R2Bm binds a small core region of the 3′UTR

**Fig. 4.**
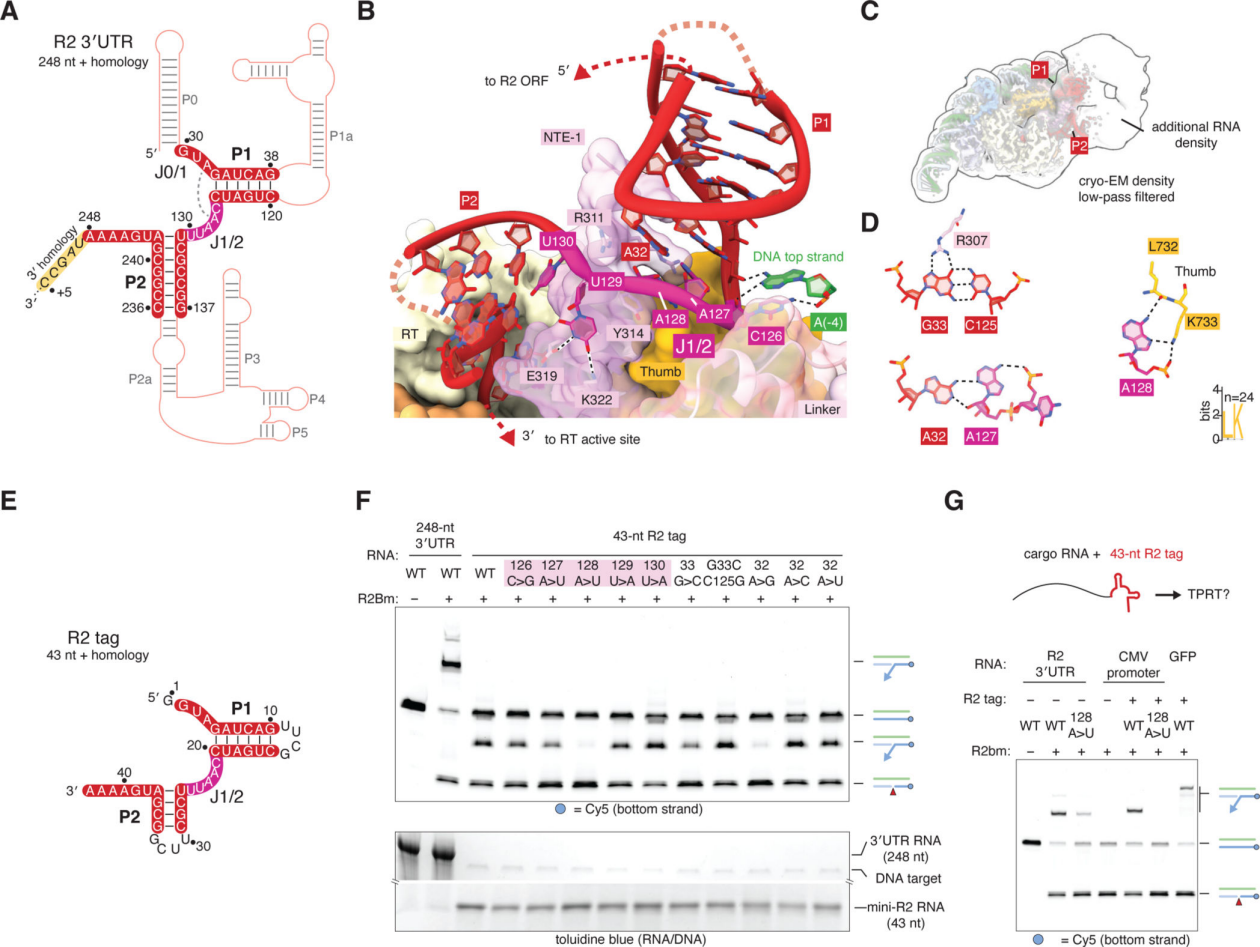
Interactions of R2Bm with the 3′ UTR RNA. (**A**) Secondary structure diagram of the 3′ UTR RNA, based on (26). Thicker strokes represent nucleotides visible in the cryo-EM density. Nucleotides are numbered from the first base of the 3′ UTR (the base following the stop codon). (**B**) Structure of the 3′ UTR RNA core and the R2Bm NTE-1 domain. Dotted lines, hydrogen bonds. (**C**) Low-pass filtered cryo-EM map. (**D**) Interactions between 3′ UTR bases. Dotted lines, hydrogen bonds. (**E**) Secondary structure of the R2 tag RNA. Unshaded bases are not in the full-length 3′ UTR. (**F**) Denaturing gel of in vitro TPRT reactions on a labeled 211-bp 28S DNA target using various R2 RNAs. Highlighted mutants are in the J1/2 region. The same gel was visualized by Cy5 fluorescence and toluidine blue staining. (**G**) The R2-tag allows TPRT of cargo RNAs. Denaturing gel shows TPRT reactions with equimolar amounts of the indicated RNAs and a labeled 211-bp 28S DNA target. R2 tag (43 nt) was added to the 3′ end of a 239-nt RNA encoding the CMV promoter or a 764-nt RNA encoding GFP.

R2Bm can only initiate TPRT on RNAs containing the R2 3′UTR (self-specificity), but the molecular basis for this is not known (25). Multiple models have been proposed for the secondary structure of the R2 3′UTR, and the divergent sequences of R2 RNAs have hindered identification of key bases (26, 27). A model for the R2 3′UTR secondary structure based on chemical probing is shown in Fig. 4A and has at least 11 stems (26). In our cryo-EM map, we resolved density for two stems and their flanking single-stranded regions (Fig. 4B). Based on nomenclature commonly used for structured RNAs, we name these stems P1 (nucleotides 33 – 38 and 120 – 135) and P2 (nucleotides 131 – 137 and 236 – 242), and term the single-stranded junction between P1 and P2 as J1/2 and the single-stranded region preceding P1 as J0/1. The rest of the 3′UTR may occupy a diffuse cloud of cryo-EM density next to these core regions (Fig. 4C).

P1 and J1/2 are mainly recognized by an ɑ helix from the R2Bm NTE-1 domain, which packs into the major groove of P1 and is wrapped by J1/2 (Fig. 4B). Arg307 recognizes the Hoogsteen edge of P1 G33, and the interaction is secured by Arg310 and Arg311. Consistently, these residues were previously shown to be essential for RNA binding (15), and the first 45 bases of the 3′UTR are essential for TPRT activity (11). J1/2 makes numerous sequence-specific contacts (Fig. 4D): A127 forms a sugar-edge pair with the Watson-Crick face of J0/1 A32 , A128 hydrogen bonds to Leu732 and Lys733 of the R2Bm thumb domain and stacks on NTE-1 Tyr314, U129 hydrogen bonds to Glu319 and Lys322 of NTE-1, and C126 stacks on and hydrogen bonds with A(−4) from the top strand of the DNA target (Fig. 4B, D).

To test if regions of the R2 3′UTR not clearly visible in the cryo-EM density are required for TPRT activity, we designed a 43-nt minimal 3′UTR – “R2 tag” – that contains only the sequences visible in the cryo-EM density, linked by tetraloops (Fig. 4E). The R2 tag was reverse transcribed as efficiently as the full 248-nt 3′UTR in a TPRT reaction. We tested the importance of the J1/2 linker by making single base transversions and found that A127U reduced activity and A128U almost completely abolished TPRT activity (Fig. 4F). Mutating G33 to C to disrupt base pairing at the bottom of stem P1 also reduced activity but could be rescued by the compensatory C125G mutation (Fig. 4F). Mutation of J0/1 A32 to G reduced activity, but mutations to C or U were tolerated. Equivalents to P1, P2, J0/1, and J1/2 can be identified in the secondary structures of diverse R2 elements (26) (fig. S7). The P1 and P2 stems have different sizes and base compositions, but positions 2 and 3 of J1/2, corresponding to A127 and A128, are conserved as adenosines, consistent with their importance for TPRT.

Because the R2 tag alone is efficiently integrated in a TPRT reaction, we tested if adding the R2 tag to the 3′ end of a “cargo” RNA would allow its integration at the 28S target site. We added the R2 tag to the 3′ end of a 239-nt CMV promoter RNA. This tagged RNA was used as efficiently as wild-type R2 3′UTR in a TPRT reaction, whereas an untagged RNA was not used, nor was an RNA tagged with R2-tag A128U mutant (Fig. 4G). A larger RNA containing the 720-nt coding sequence for GFP and a 3′ R2 tag was also reverse transcribed in a TPRT reaction (Fig. 4G).

### R2Bm can be retargeted with CRISPR-Cas9

**Fig. 5.**
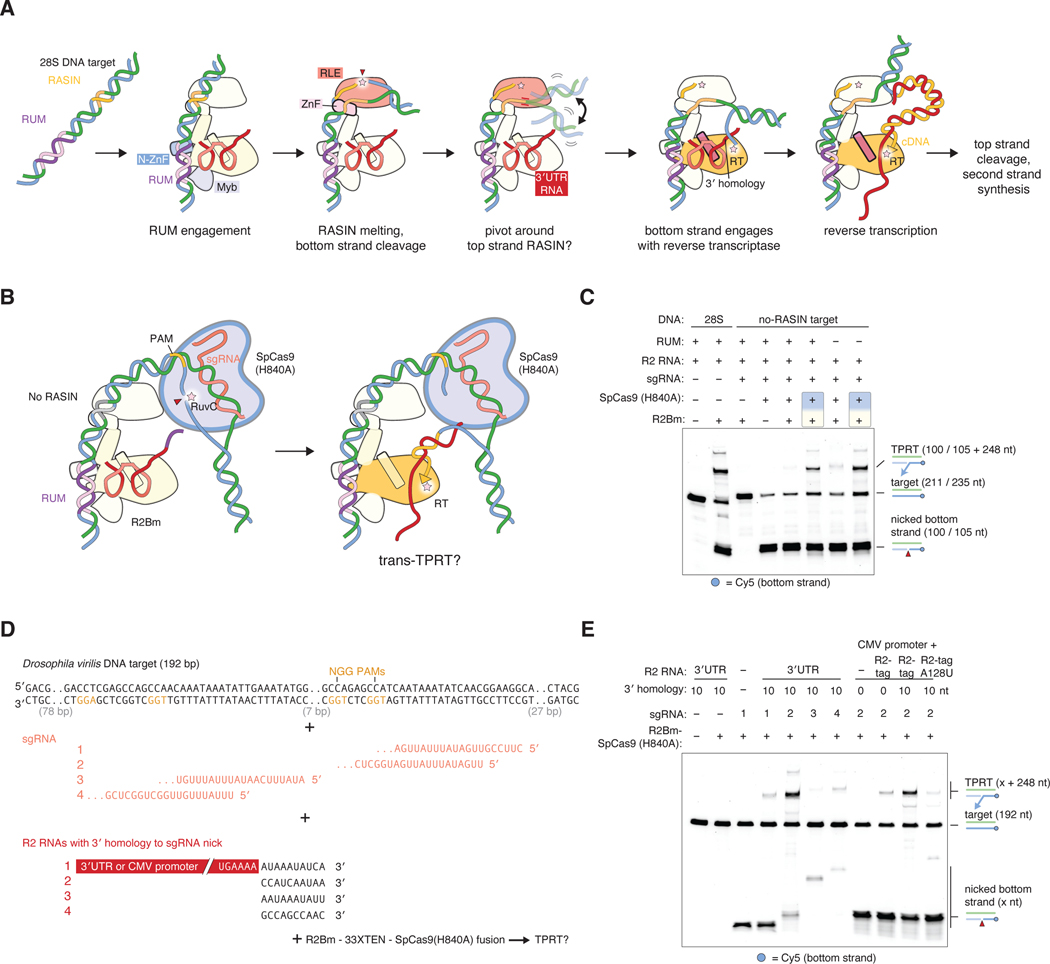
Mechanism and retargeting of first strand synthesis by R2Bm. (**A**) Model for the initial stages of target site cleavage and first strand synthesis. (**B**) Design of R2Bm + Cas9 experiments. (**C**) Complementation of DNA target site mutants by Cas9 cleavage in *trans* and *cis*. The denaturing gel shows in vitro TPRT reactions on a labeled 211-bp target corresponding to the wild-type 28S target, or two 235-bp targets: one where the RASIN TAAGGTA is replaced by 31 bp of unrelated sequence, and another where the 13-bp RUM is additionally scrambled. R2Bm and SpCas9(H840A) were added in *trans*, or in *cis* connected by a 33XTEN linker ( fusion indicated by a shaded box). The sgRNA is complementary to the inserted sequence and nicks 40 nt from the last RUM base. The R2 RNA is the 3′ UTR with 5 nt of 3′ homology to the nick site. (**D**) Sequences used for retargeting R2Bm to an unrelated locus from the *Drosophila virilis* genome. (**E**) Denaturing gel of in vitro TPRT reactions on the labeled 192-bp *Drosophila virilis* target. sgRNAs are numbered as in (D); all R2 RNAs or R2-tagged RNAs have 10 nt of 3′ homology to the nick site of the sgRNA.

Our structural and biochemical observations suggest a multi-step model for initiation of TPRT: the R2Bm N-terminal domains first detect a RUM sequence, followed by cleavage of the bottom strand at the RASIN site, possible pivoting of the nick around the top strand into the RT active site, annealing of any 3′ homology to the nicked bottom strand, and finally initiation of reverse transcription (Fig. 5A). This model implies that R2Bm could prime reverse transcription off an exogenously nicked bottom strand close to the R2Bm binding site (Fig. 5B). To test this, we replaced the RASIN and downstream sequences of the 28S DNA target with an unrelated sequence containing an efficient SpCas9 target sequence, but kept the RUM sequence to anchor R2Bm (Fig. 5B). This substrate could not be cleaved by R2Bm, but was nicked efficiently by a SpCas9 H840A nickase mutant (Fig. 5C). When SpCas9 and R2Bm were added together with a single-guide RNA (sgRNA) and an R2 3′UTR RNA with 5 nt of 3′ homology to the sgRNA nick site, we detected low amounts of TPRT activity. This activity was enhanced when the R2Bm and SpCas9 proteins were fused with a 33XTEN flexible linker (Fig. 5C). The RUM was not required for Cas9-directed TPRT, as mutating the RUM did not reduce activity (Fig. 5C). This suggests Cas9 might be able to direct R2Bm to perform TPRT at loci other than the 28S target. We mixed the R2Bm-Cas9(H840A) fusion protein with a 192-bp target sequence from *Drosophila virilis*, various sgRNAs, and R2 3′UTRs with 10 nt of 3′ homology to the nick site dictated by the sgRNA (Fig. 5D). We found TPRT activity at all Cas9 nick sites, with one sgRNA (guide 2) giving efficient activity (Fig. 5E). Adding R2Bm and SpCas9(H840A) as separate polypeptides also yielded efficient TPRT with guide 2, but was less robust with other guides (fig. S9). The 239-nt CMV promoter RNA with a 3′ R2 tag and 10 nt of homology to the guide 2 nick site was also reverse transcribed efficiently; this activity required the R2 tag and was reduced in the absence of 3′ homology or with the R2 tag A128U mutation (Fig. 5E). Larger RNAs like GFP could also be reverse transcribed at the guide 2 nick site (fig. S9). In summary, R2Bm can be retargeted by Cas9 to perform TPRT at unrelated loci, and the R2 tag can direct incorporation of cargo RNAs at these sites.

## Discussion

Here we show the structure of a non-LTR retrotransposon during transposition, and we dissect the principles of target DNA and self-RNA recognition. Our structure suggests that R2Bm uses its N-ZnF and Myb domains to locate the endonuclease target sequence, a model that contrasts with the model for other non-LTR retrotransposons where the endonuclease domain is the only determinant of target site selection (28, 29). We identified two essential target site motifs - the RUM and RASIN - that are recognized by R2Bm, but we note that searching the *B. mori* genome with a RUM-RASIN consensus motif yields many potential off-target sites outside of the ribosomal DNA arrays (fig. S10). We examined the sequence of a previously identified *B. mori* non-28S insertion in (30) and found the target site had limited similarity with 28S but had a TTAAcG|T RASIN motif (‘|’ indicates insertion site, lower-case is deviation from 28S) and a GCTACTTGCGCAT RUM the correct distance upstream of the RASIN (fig. S10). Non-28S insertions however are rare, and so it is likely other factors are important in regulating R2Bm transposition, including chromatin accessibility, other sequence motifs, or the ability of the target DNA to bend and melt.

Non-LTR retrotransposons form a diverse family, and even within the R2 superclade there are notable differences between elements. R2Bm is a representative of the R2-D clade of elements, which have a single C2H2 N-terminal ZnF domain, but R2-A clade elements have three tandem N-terminal ZnF domains (31) that may create a more extensive DNA-binding interface with greater stringency in target site selection. More broadly, non-LTR retrotransposons can be divided into two types based on their endonuclease domains: those that like R2Bm use a C-terminal restriction enzyme-like (RLE) domain, and those that, like human LINE-1, use an unrelated N-terminal apurinic/apyrimidinic endonuclease (APE) domain (32, 33). Structure prediction using AlphaFold (34) suggests that, in these retrotransposons, the APE domain has a distinct position to the RLE domain in R2Bm, suggesting there may be mechanistic differences in how target cleavage is coupled to reverse transcription (fig. S5) (35). Nonetheless, the similarity between the DNA interface on the R2Bm thumb domain and the corresponding interface in the group IIC intron (fig. S5) suggests this interface might be conserved amongst most non-LTR retrotransposons (19). Indeed, the upstream DNA from R2Bm was easily modeled into an AlphaFold model of human LINE-1 ORF2, including the thumb interactions but also strand separation by the CCHC ZnF domain, which in LINE-1 ORF2 corresponds to the C-terminal cysteine-rich domain (fig. S5).

Overall, the results of this work advance our understanding of transposition by non-LTR retrotransposons and suggest avenues for engineering these transposons for targeted gene insertions.

## Supplementary Material

SI

## Data Availability

The cryo-EM map has been deposited in the Electron Microscopy Data Bank with accession code EMD-40033. The coordinates for the atomic model have been deposited in the Protein Data Bank with accession code 8GH6. The raw cryo-EM data have been deposited in EMPIAR with accession code EMPIAR-11458.
