# Supplementary material for: Structure of the R2 non-LTR retrotransposon initiating target-primed reverse transcription
Source: Science. Author manuscript; Available in PMC 2023 Sep 13. (PMC10499050; doi:10.1126/science.adg7883)
Supplement: SI [file NIHMS1900614-supplement-SI.pdf]

Supplementary Materials for

Structure of the R2 non-LTR retrotransposon initiating target-primed reverse transcription

Max E. Wilkinson, Chris J. Frangieh, Rhiannon K. Macrae, and Feng Zhang.

Correspondence to: zhang@broadinstitute.org

This PDF file includes:

Materials and Methods
Figs. S1 to S10
Tables S1 to S3

Materials and Methods

Cloning

Residues 111-1114 of the R2Bm ORF (as numbered in UniProt accession V9H052) were cloned by Gibson Assembly into a pET bacterial expression plasmid that adds an N-terminal 14xHis – MBP – bdSUMO tag and a C-terminal Twin-StrepII tag. The R2Bm ΔN, ΔRT6a, and RD>AA mutants were generated from this plasmid by KLD cloning (New England Biolabs). The R2BmΔN mutant maintains the His-MBP-bdSUMO tag and then starts at residue Ala288 of R2Bm. The ΔRT6a mutant changes residues P_{670}DGHRKKHHYLT$_{681}$ to PGGHYLT. The RD>AA mutant changes $R_{901}D_{902}$ to AA. The R2Bm-Cas9 fusion was made by Gibson Assembly. A 33XTEN linker was added after Gly_{1114} (SGGSSGGSSGSETPGTSESATPESSGGSSGGSS) followed by *Streptococcus pyogenes* Cas9 (H840A) and a C-terminal Twin-StrepII tag. The 200 bp flanking the R2Bm 28S target site were cloned into a pACYC backbone. The R2Bm 3′UTR was cloned into a pRS426 backbone. Plasmid sequences were verified by Tn5 tagmentation and high-throughput sequencing, as previously described (*36*). Plasmid sequences can be seen in Table S2.

Protein purification

All R2Bm constructs were expressed and purified in the same manner: the expression plasmid was transformed into *E. coli* BL21(DE3) (New England Biolabs) and grown at 37°C in Terrific Broth supplemented with 25 mM disodium hydrogen phosphate, 25 mM potassium dihydrogen phosphate, 50 mM ammonium chloride, 5 mM sodium sulfate, 0.5% (w/v) glycerol, 0.2% (w/v) α-lactose monohydrate, 0.05% (w/v) glucose, 2 mM magnesium chloride (TB-based autoinduction media), and 50 µg/L ampicillin. The temperature was reduced to 22°C during mid-log phase, and cells were grown for another 16 – 20 hr. Cells were harvested and resuspended in R2 Lysis Buffer (50 mM Tris-HCl pH 7.4, 1 M NaCl, 10% glycerol, 5 mM beta-mercaptoethanol) supplemented with EDTA-free cOmplete protease inhibitor (Roche). Cells were lysed using a LM20 microfluidizer device (Microfluidics), and cleared lysate was bound to Strep-Tactin Superflow Plus resin (Qiagen). Resin was washed first with lysis buffer and then with R2 Storage Buffer (20 mM HEPES-KOH pH 7.9, 500 mM KCl, 10% glycerol) before elution with R2 Storage Buffer supplemented with 5 mM desthiobiotin. Fractions containing protein were concentrated in a Vivaspin 20 centrifugal concentrator (50000 MWCO; Sartorius) to 1.5 mg/mL ($OD_{280\ nm}$ = 1.8) for the wild-type R2Bm, 1.9 mg/mL ($OD_{280\ nm}$ = 2.4) for the ΔN mutant, 1.4 mg/mL ($OD_{280\ nm}$ = 1.7) for the ΔRT6a mutant, 0.9 mg/mL ($OD_{280\ nm}$ = 1.1) for the RD>AA mutant, and 0.6 mg/mL ($OD_{280\ nm}$ = 0.6) for the R2Bm-SpCas9(H840A) fusion. Protein concentrations were normalized by densitometry after SDS-PAGE and Coomassie blue staining. Proteins were flash frozen in liquid nitrogen and stored at -80°C.

RNA in vitro transcription

Templates for in vitro transcription (IVT) were produced by PCR with a T7 promoter added to the forward primer. For biochemical experiments, PCR reactions were diluted 1/10 in the IVT reaction mixture which contained 4 mM ATP, 4 mM CTP, 4 mM GTP, 4 mM UTP, 20 mM $MgCl_2$, 40 mM Tris-HCl pH 8.0, 10 mM DTT, 1 mM spermidine, and 85 µg/mL of homemade T7 RNA polymerase, and then incubated at 37°C for 90 minutes. The pyrophosphate precipitate was pelleted and removed, and reactions were treated with 1/100 volume of RNase-free DNase I (New England Biolabs) at 37°C for 15 minutes. RNA was purified using 1.4 volumes of SPRIselect paramagnetic beads (Beckman Coulter) and dissolved in water. RNAs smaller than

100 nt (sgRNAs and R2-tags) were purified using 3 volumes of SPRIselect beads and 3.33 volumes of isopropanol. RNA concentrations were determined using a Nanodrop (Thermo Fisher Scientific) and then normalized by densitometry after denaturing PAGE and toluidine blue staining. RNA for the cryo-EM complex was produced similarly, except the PCR product was purified using SPRIselect beads, and the IVT reaction was not DNase treated or purified and was used directly for complex formation. RNA sequences can be found in Table S3.

Preparation of DNA substrates

210-bp DNA targets were prepared by PCR with Phusion Flash polymerase (Thermo Scientific) using a plasmid containing the 28S rRNA gene sequence as a template. The forward primer for the top strand had a 5′ fluorescein label and PvuII site (sequence /56-FAM/TTTTTCAGCTGGTTGACGCGATGTGATTTCTG) and the reverse primer for the bottom strand had a 5′ Cy5 label (sequence /5Cy5/TTCCCTTGGCTGTGGTTTCG). PCR products were purified with 1.4 volumes of SPRIselect paramagnetic beads (Beckman Coulter) and dissolved in water. 76-bp DNA targets were prepared by annealing labeled oligos synthesized by IDT. The top strand sequence is /5BiosG/TTTCAGCTGTGAAGCGCGGGTAAACGGCGGGAGTAACTATGACTCTCTTAAGGTAGCCAAATGCCTCGTCATCTAA. The bottom strand sequence is /56-FAM/TTAGATGACGAGGCATTTGGCTACCTTAAGAGAGTCATAGTTACTCCCGCCGTTTACCCGCGCTTCACAGCTGAAA. Equal volumes of 100 µM top and 100 µM bottom strands were mixed in 10 mM HEPES-KOH pH 7.9 and 60 mM KCl, heated to 95°C for 2 min then gradually cooled to 25°C over 45 min using a thermocycler. These 50 µM annealed substrates were stored at -20°C until use. For the experiment in Figure 3E, this 76 bp bottom strand was annealed with “17d” AAATGCCTCGTCATCTA, “32u” GCGGGTAAACGGCGGGAGTAACTATGACTCTC, or “60” GCGGGTAAACGGCGGGAGTAACTATGACTCTCTTAAGGTAGCCAAATGCCTCGTCATCTA.

In vitro TPRT reactions

TPRT reactions contained 20 nM labeled DNA substrate, 1 µM 3′UTR RNA, and 210 nM R2Bm protein in a reaction buffer containing 20 mM HEPES-KOH pH 7.9, 400 mM potassium acetate, 5 mM magnesium acetate, and 25 µM of each dNTP. Reactions were incubated at 37°C for 30 min and stopped with 1 volume of 2x TBE-urea sample buffer (90 mM Tris base, 90 mM boric acid, 2 mM EDTA, 12% Ficoll Type 400, 7 M urea, and 0.02% bromophenol blue) supplemented with 1 µg RNase A per reaction. Reactions were boiled at 95°C for 150 sec, placed on ice, and run on a precast 10% acrylamide TBE-Urea gel (Invitrogen) at 400 V for 12 – 15 min. Gels were visualized using a ChemiDoc (Bio-Rad).

R2 cleavage assay for determination of the Retrotransposon Upstream Motif (RUM)

A target library was ordered from IDT as a single-stranded oligonucleotide (/5BiosG/AGATGACGAGGCATTTGGCTACCTTAACTCTACGCCGCAACGNNNNNNNNNNNNNAGCTAGNNNNNNCTGTCTCTTATACACATCTGACGCTGCCGACGA) containing the Illumina R1 primer site (TCGTCGGCAGCGTCAGATGTGTATAAGAGACAG), an upstream 6-bp unique molecular identifier (UMI, NNNNNN), spacer sequence (AGCTAG), 13*N RUM motif, scrambled sequence (CGTTGCGGCGTAGAG), and RASIN motif. The single-stranded oligonucleotide was converted to a double-stranded target library via an 8-cycle

PCR with a forward primer (TCGTCGGCAGCGTCAGATG) and a reverse primer (GCTGAGGCTACCTTAACTCTACGCCGC) that also added the Nt.BbvCI cleavage site (CCTCAGC). The target library was first digested with R2 protein for 30 min under the conditions described above and purified. The purified reaction products were digested at 37°C for 2 hr with Nt.BbvCI nicking endonuclease (New England Biolabs). A sticky end sequencing adapter was made by PNK treatment of an oligonucleotide containing the Illumina R2 sequence (CTGTCTCTTATACACATCTCCGAGCCCACGAGAC) followed by annealing with an oligonucleotide (GTCTCGTGGGCTCGGAGATGTGTATAAGAGACAGggcta) containing the Illumina R2 sequence and overhang complementary to the Nt.BbvCI nick site, and the sticky end sequencing adapter was ligated to the digested library with T4 DNA ligase at 16°C for 15 hr (New England Biolabs). NGS libraries were prepared by PCR with a forward primer (AATGATACGGCGACCACCGAGATCTACACAAGTAGAGTCGTCGGCAGCGTCAGATG TGTA) and a reverse primer (CAAGCAGAAGACGGCATACGAGATCATGATCGGTCTCGTGGGCTCGGAGATGTGT) by KAPA HiFi polymerase (Roche) to add Illumina P5, P7, i5, and i7 sequences. NGS libraries were sequenced on two Illumina NextSeq 500 sequencers with 46 cycles read 1, 8 cycles index 1, 8 cycles index 2, and 26 cycles read 2. Sequencing output was filtered to reads containing the expected spacer, scrambled, and RASIN sequences. A cleaved RUM was defined as any RUM with more than 20 UMIs. All cleaved RUMs were combined for calculation of the weblogo using WebLogo 3.7.12 (*37*).

NGS of TPRT products

In vitro TPRT reactions were performed as described above on a DNA substrate containing a biotinylated bottom strand. Reaction products were purified and RNAse treated followed by denaturation in TBE-urea sample buffer (90 mM Tris base, 90 mM boric acid, 2 mM EDTA, 12% Ficoll Type 400, 7 M urea, and 0.02% bromophenol blue) and size selection via extraction from a precast 10% acrylamide TBE-Urea gel (Invitrogen) to isolate extended bottom strand products. Purified bottom strand extension products were incubated with Dynabeads M-270 Streptavidin (Thermo Fisher Scientific) in 1x binding buffer (5 mM Tris-HCl pH 7.5, 0.5 mM EDTA, 1 M NaCl) with agitation (1100 rpm) at room temperature for 30 min. After washing, a 5′ pre-adenylated and 3′ capped ssDNA oligo containing the Illumina R2 sequence (/5rApp/CTGTCTCTTATACACATCTCCGAGCCCACGAGAC/3SpC3/, Integrated DNA Technologies) was incubated with the beads and a thermostable ssDNA ligase (New England Biolabs) for 16 hr at 65°C with agitation (1100 rpm). Washed beads were input into a PCR with a forward primer annealing to the 3′ end of the 28S target sequence containing Illumina P5 and i5 sequences (AATGATACGGCGACCACCGAGATCTACACCATGCTTATCGTCGGCAGCGTCAGATG TGTATAAGAGACAGttcccttggctgtggtttcg) and a reverse primer containing Illumina P7 and i7 sequences annealing to the single-stranded adapter ligated to the 3′ end of the immobilized extension products (CAAGCAGAAGACGGCATACGAGATCATGATCGGTCTCGTGGGCTCGGAGATGTGT). NGS libraries were sequenced on an Illumina MiSeq with 120 cycles read 1, 8 cycles index 1, 8 cycles index 2, and 120 cycles read 2.

In vitro Cas9-directed TPRT reactions

Cas9-directed TPRT reactions used the same conditions as above, except for containing 1.5 µM 3′UTR RNA, 1.5 µM sgRNA; 100 nM R2Bm protein, 1.5 µM SpCas9 H840A nickase (Alt-R V3, Integrated DNA Technologies), or 100 nM of their fusion. After reaction completion, reactions were treated with 0.8 U Proteinase K (New England Biolabs) and 1 µg RNase A before electrophoresis as above. The "no-RASIN" target in Fig. 5C has the following sequence: TTTTTCAGCTGGTTGACGCGATGTGATTTCTGCCCAGTGCTCTGAATGTCAAAGTGAAGAAATTCAATGAAGCGCGGGTAAACGGCGGGAGTAACTATGACTCTCTAATACCCCATAACAACAACCCCCTAATCAACGCCAAATGCCTCGTCATCTAATTAGTGACGCGCATGAATGGATTAACGAGATTCCCACTGTCCCTATCTACTATCTAGCGAAACCACAGCCAAGGGAA. The sgRNA for nicking this target has the protospacer sequence GGCATTTGGCGTTGATTAGG. The *Drosophila virilis* target has the following sequence: GACGGTTTGCCGATGTGCAACCGAAATATATCGGAAGAGAATTGAATAAAATTGTTTTTCATTGTTTGTTTTAACAAACTCGGACCTCGAGCCAGCCAACAAATAAATATTGAAATATGGAAAGGTCGCCAGAGCCATCAATAAATATCAACGGAAGGCACGCCGTATGCACAGCAACCAACATGAGCTACG. sgRNAs 1, 2, 3, 4 have the following protospacer sequences: CTTCCGTTGATATTTATTGA, TTGATATTTATTGATGGCTC, ATATTTCAATATTTATTTGT, TTTATTTGTTGGCTGGCTCG.

R2 complex formation and purification for cryo-EM

R2 complex formation used a 1 mL TPRT reaction containing 160 nM of a 76-bp 28S DNA target with a 5′ fluorescein label on the bottom strand and a 5′ biotinylated top strand, 210 nM of His-MBP-SUMO-tagged R2Bm protein, 25 µM of 2′,3′-dideoxythymidine, 0.4 volumes of 3′UTR+5 nt 3′ homology IVT reaction, and 1 µg/mL bdSENP1 protease (to remove the His-MBP-SUMO tag during complex formation) in a reaction buffer containing final concentrations 20 mM HEPES-KOH pH 7.9, 400 mM potassium acetate, 5 mM magnesium acetate, 1.6 mM ATP, 1.6 mM CTP, 1.6 mM GTP, 1.6 mM UTP, 8 mM $MgCl_2$, 16 mM Tris-HCl pH 8.0, 4 mM DTT, 0.4 mM spermidine, and 35 µg/mL T7 RNA polymerase.

The reaction was incubated at 37°C for 40 min before incubation with Streptavidin Sepharose High Performance resin (Cytiva) for 40 min at 4°C. The resin was washed with R2 buffer (20 mM HEPES-KOH pH 7.9, 500 mM potassium acetate, 5 mM magnesium acetate, 1 mM TCEP), before washing with R2 buffer containing 5 mM desthiobiotin and then eluting at 37°C for 30 min with R2 buffer containing 5 mM desthiobiotin and 2 µL (20 units) PvuII restriction enzyme (New England Biolabs). Eluted complexes were concentrated with a 30,000 MWCO Amicon Ultra 0.5 mL centrifugal filter (Millipore-Sigma) and then diluted to $OD_{260\ nm}$ = 3 in R2 buffer and 0.1 mM dTTP.

For cryo-EM grid preparation, a freshly glow-discharged (12 s at 25 mA) Cu300 R2/2 holey carbon grid with a 2-nm layer of amorphous carbon (Quantifoil) was mounted in the chamber of a Vitrobot Mark IV (Thermo Fisher Scientific) maintained at 12°C and 100% humidity. 3 µL of R2 complex was applied and after 30 sec was manually blotted using Ø55 grade 595 filter paper (Ted Pella) and plunged into liquid ethane.

Cryo-EM data collection

Cryo-EM data were collected using the Thermo Scientific Titan Krios G3i cryo TEM at MIT.nano using a K3 direct detector (Gatan) operated in super-resolution mode with 2-fold binning, and an energy filter with slit width of 20 eV. Micrographs were collected automatically using EPU in AFIS mode, yielding 16,551 movies at 130,000x magnification with a real pixel

size of 0.663 Å, with defocus ranging from -1.5 μm to -2.5 μm with an exposure time of 0.69 s, fractionated into 40 frames and a flux of 26.9 e^-/pix/s giving a total fluence per micrograph of 42.2 $e^-/Å^2$.

Cryo-EM data processing

All cryo-EM data were processed using RELION-4.0 (38) (Figure S3). Movies were corrected for motion using the RELION implementation of MotionCor2, with 4x6 patches and dose-weighting. CTF parameters were estimated using CTFFIND-4.1. Particle picking was first carried out using Topaz with the general model (39), yielding 1,085,471 particles. One round of 3D classification, using an orientation-biased initial model from previous datasets was used to select 235,212 promising particles, which were subject to two rounds of 2D classification to yield 32,271 particles with high quality 2D classes, which were used to train a Topaz picking model.

The full dataset was then picked again using this new model, yielding 1,716,620 particles that were first filtered by 2D classification to 689,524 particles, then 3D classification to 383,198 particles which were then polished and refined to 3.12 Å resolution. However, the map showed significant artifacts, so was subject to 3D classification without alignment, using a mask around the core, 40 iterations, and a regularization parameter "T" of 4. This yielded a small subset of 39,616 particles with sharp features, which were refined to 3.20 Å resolution. Per-particle defocus was then refined, followed by refinement of anisotropic magnification, beam tilt, trefoil, and 4th order aberrations. A final refinement produced an isotropic map at 3.08 Å resolution free of streaking artifacts and with features consistent with the estimated resolution. Resolution is reported using the gold-standard Fourier Shell Correlation with 0.143 cutoff.

Model building

An initial model for R2Bm ORF was generated automatically by ModelAngelo (40), which was adjusted manually, with some loops filled using Coot (41). Coot was used to build the 3′UTR RNA and target DNA de novo. The model was refined first using ISOLDE (42) then with PHENIX (43), just performing one macro-cycle of global minimization and ADP refinement and skipping local grid searches. Figures were generated using UCSF ChimeraX (44).

Data availability

The cryo-EM map has been deposited in the Electron Microscopy Data Bank with accession code EMD-40033. The coordinates of the atomic model have been deposited in the Protein Data Bank under accession code 8GH6. The raw cryo-EM micrographs are available at EMPIAR with accession number EMPIAR-11458.

Fig. S1

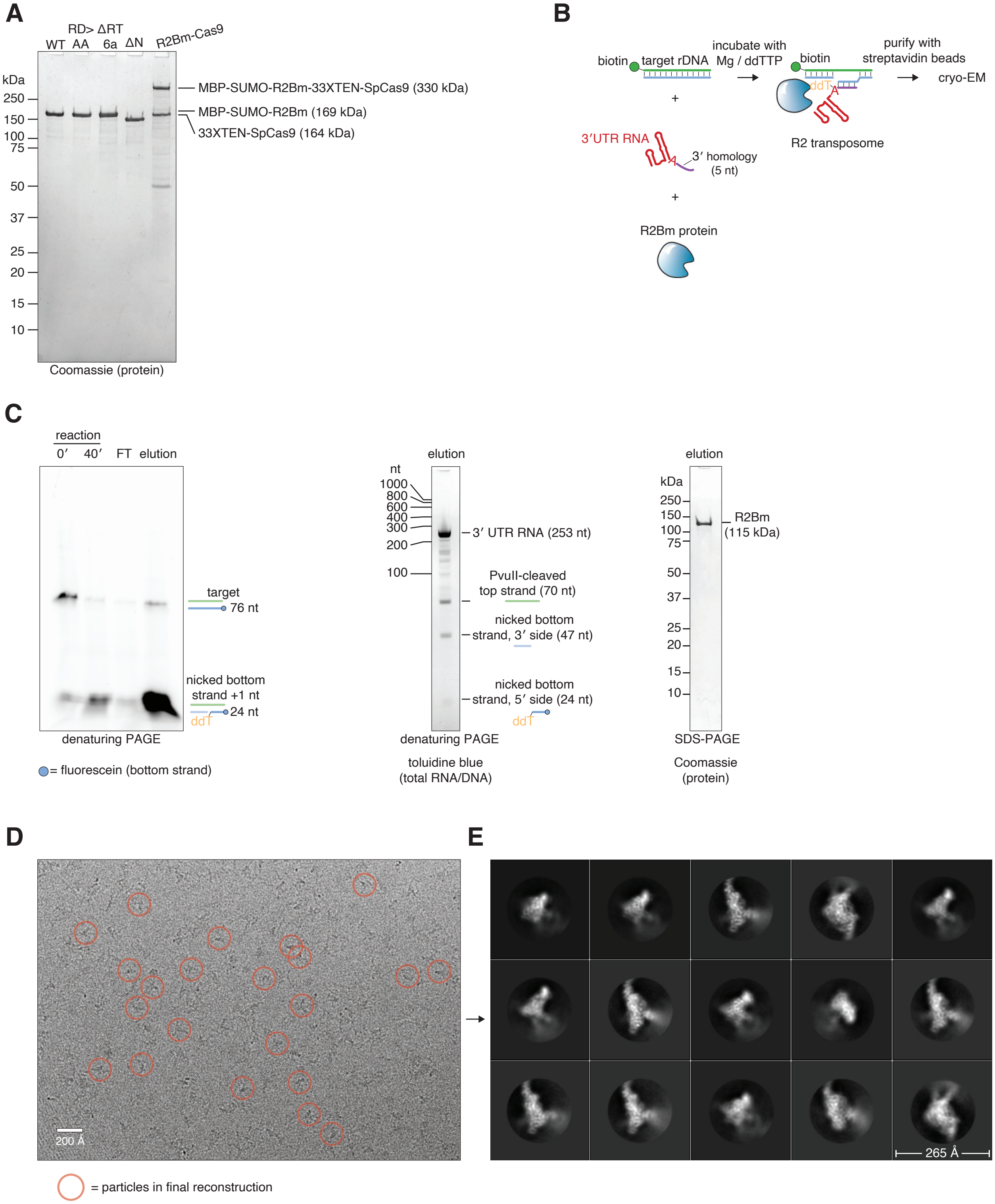

Fig. S1. Purification of R2Bm and its TPRT complex. (**A**) SDS-PAGE gel of purified R2Bm proteins and mutants. (**B**) Strategy for purifying the R2Bm TPRT complex. (**C**) Purification of the R2Bm TPRT complex. The first gel shows the input stalled TPRT reaction. FT, flowthrough from streptavidin beads. Nucleic acids from the purified complex were phenol-chloroform

extracted and ethanol precipitated before running on the second gel. (**D**) Example cryo-EM micrograph of the purified R2Bm TPRT complex. (**E**) 2D class averages of the R2Bm TPRT complex.

Fig. S2

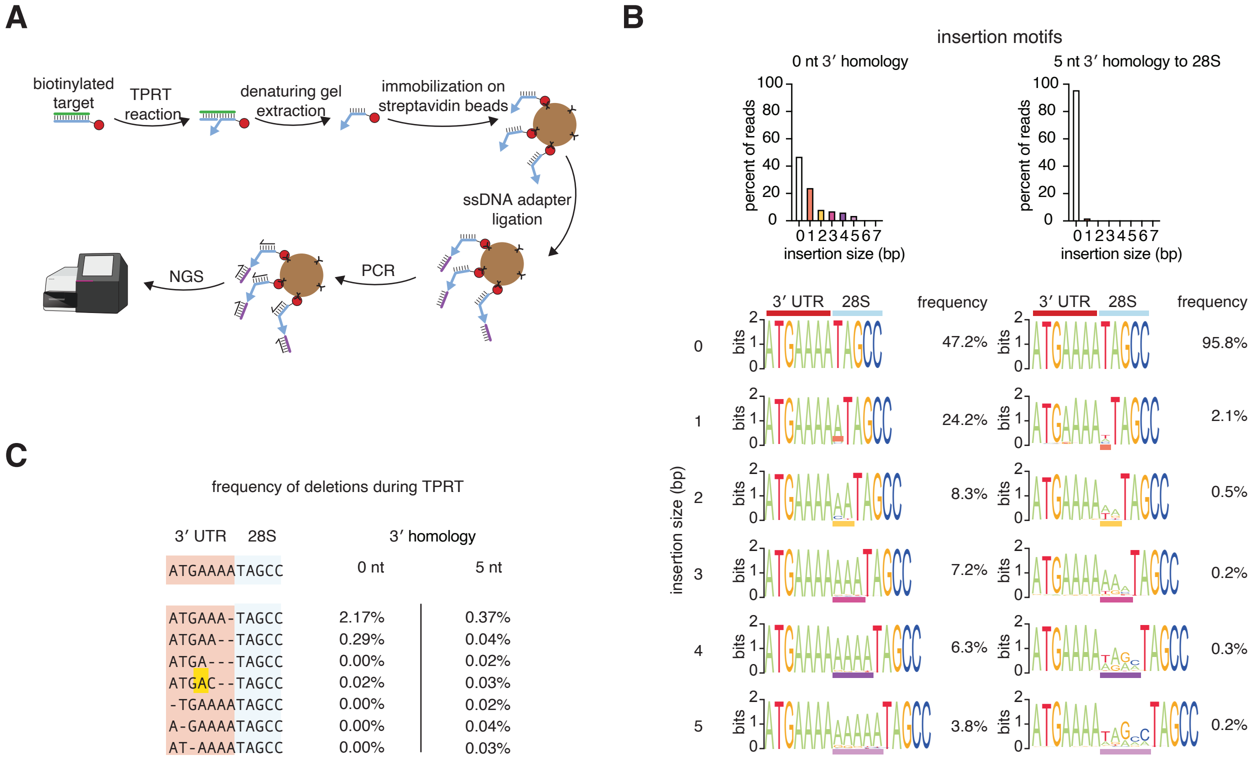

Fig. S2. Sequencing of TPRT reaction products. (**A**) Schematic for preparation of sequencing libraries from TPRT reaction products. (**B**) Non-templated insertions at the TPRT site are more frequent with no 3′ homology. Histograms are shown for insertion sizes with and without 5 nt 3′ homology, and for each insertion size a sequence logo is shown for the insertions. Insertions for RNAs with no 3′ homology are A-rich, whereas the rare insertions for RNAs with 5 nt 3′ homology appear to resemble the homology itself, implying rare cases of initiating TPRT at the very 3′ end of the RNA even with homology. (**C**) Examples of deletions during TPRT. These could arise from TPRT initiating upstream of the RNA 3′ end, or skipping template nucleotides during reverse transcription, or from heterogeneity in the supplied RNA.

Fig. S3

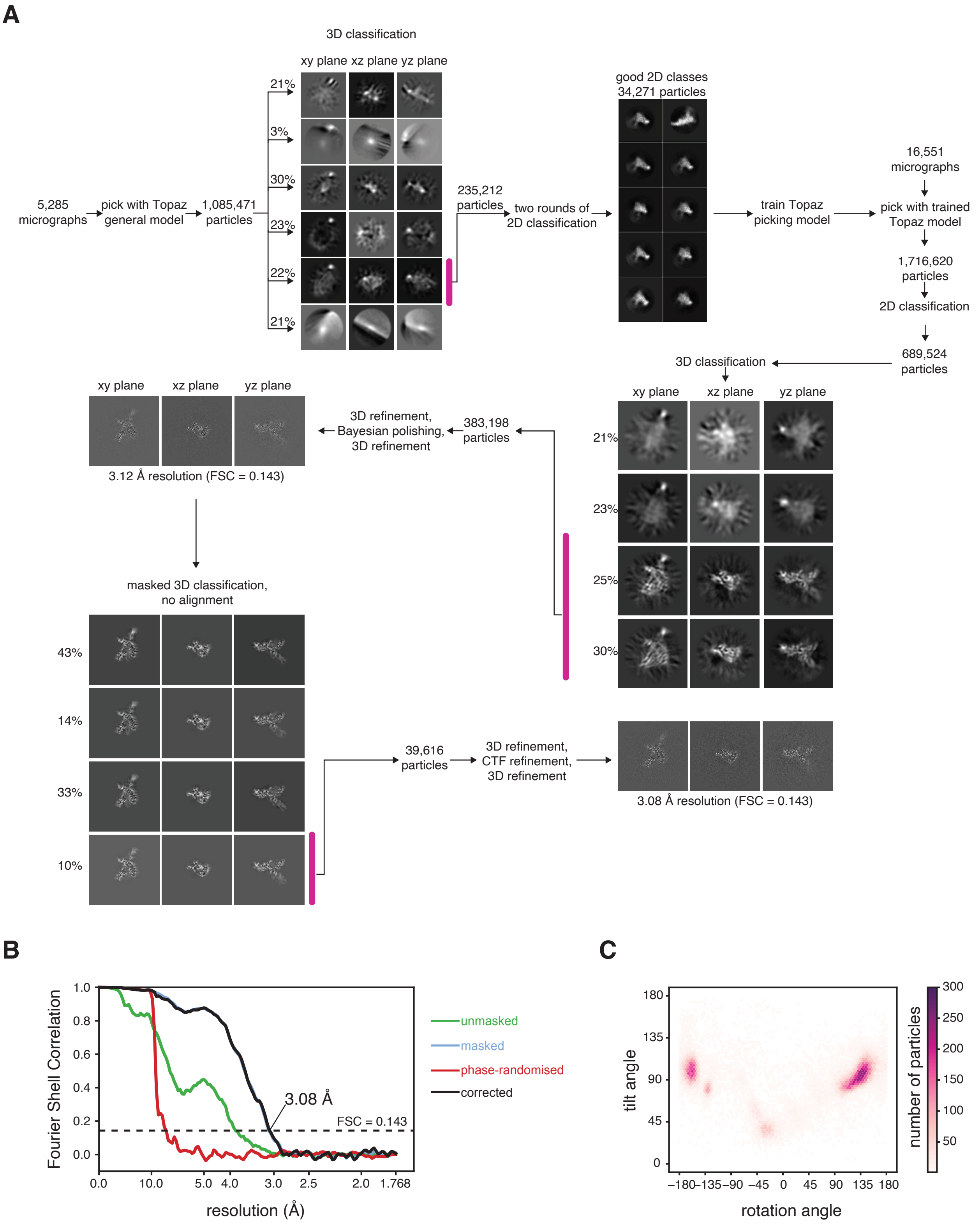

Fig. S3. Cryo-EM data processing. (**A**) Flowchart outlining how cryo-EM data were processed. Three central slices are shown for each 3D map. (**B**) Gold-standard Fourier Shell Correlation curve for the final reconstruction. (**C**) Orientation distribution plot for the final reconstruction.

Fig. S4

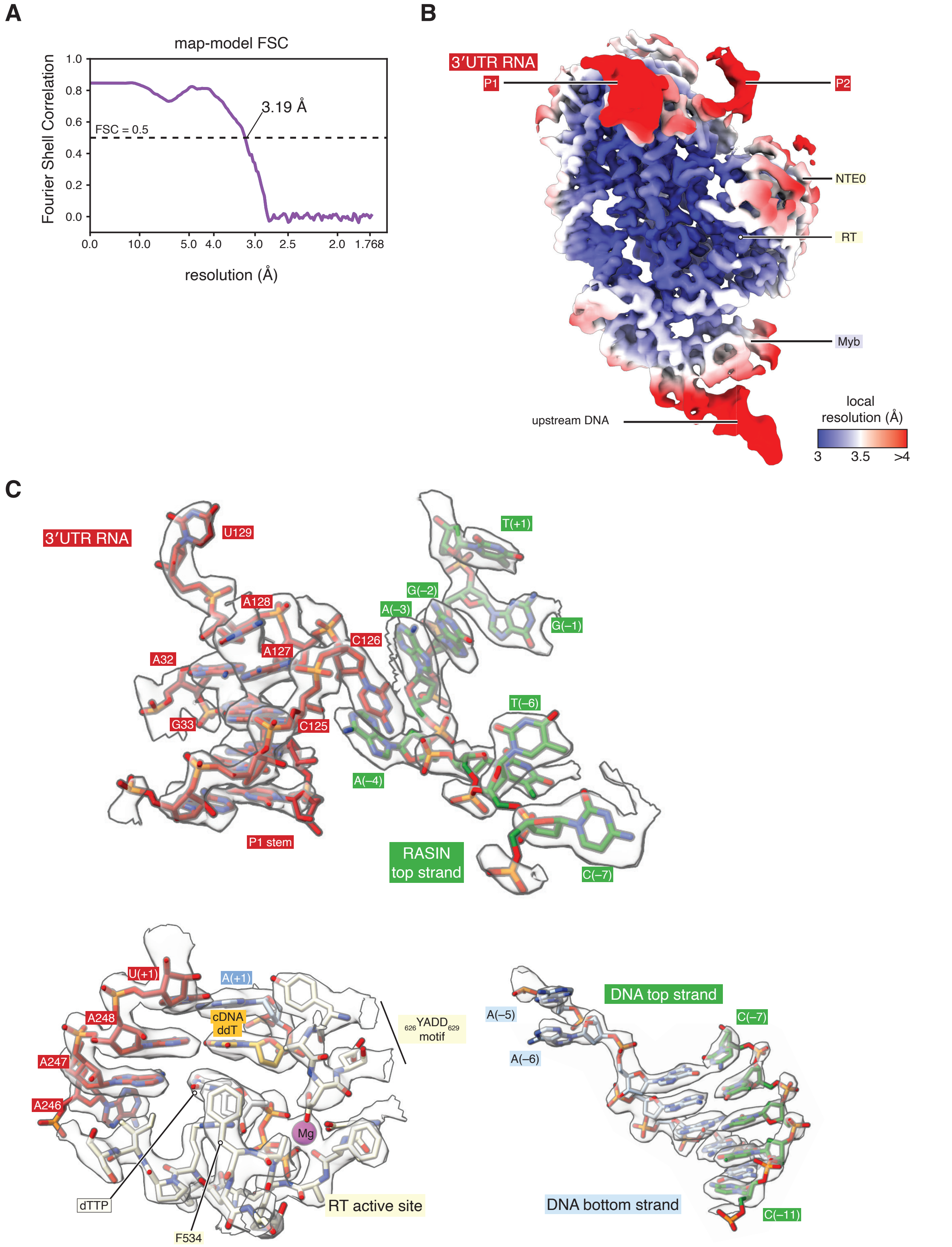
A
map-model FSC
Fourier Shell Correlation
1.0
0.8
0.6
0.4
0.2
0.0
FSC = 0.5
3.19 Å
0.0
10.0
5.0
4.0
3.0
2.5
2.0
1.768
resolution (Å)
B
3′UTR RNA
P1
P2
NTE0
RT
Myb
upstream DNA
local resolution (Å)
3
3.5
>4
C
3′UTR RNA
U129
A128
A127
C126
A32
G33
C125
P1 stem
T(+1)
G(−2)
A(−3)
G(−1)
T(−6)
A(−4)
RASIN top strand
C(−7)
U(+1)
A(+1)
A248
cDNA ddT
626YADD629 motif
A247
A246
Mg
dTTP
F534
RT active site
DNA top strand
A(−5)
C(−7)
A(−6)
DNA bottom strand
C(−11)

Fig. S4. Fit of the model to the cryo-EM map. (**A**) Map-to-model Fourier Shell Correlation as calculated in PHENIX, softly masking the map around the fitted model. (**B**) Unsharpened cryo-EM map coloured by local resolution with RELION. (**C**) Example cryo-EM densities for different parts of the structure.

Fig. S5

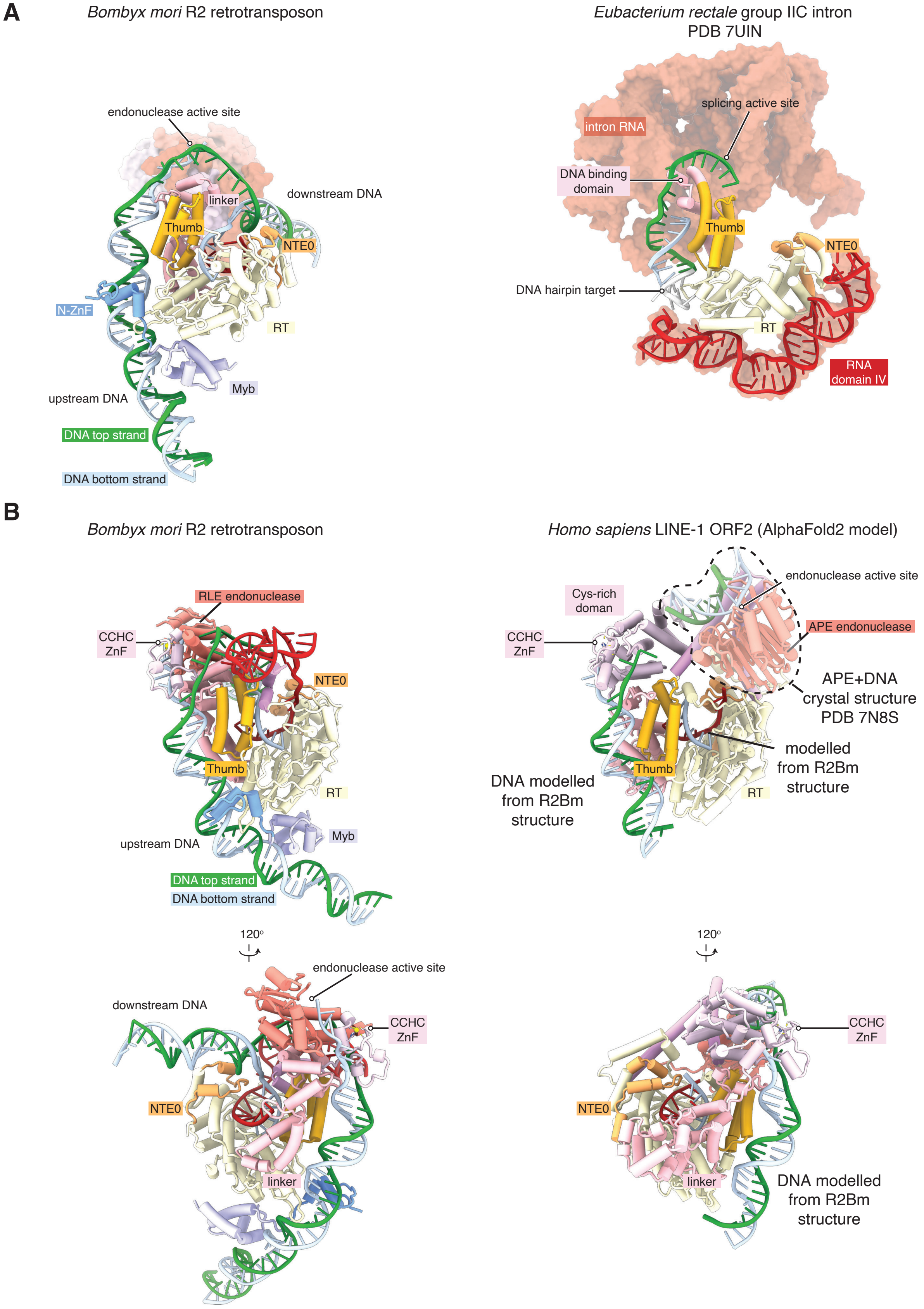
A
Bombyx mori R2 retrotransposon
endonuclease active site
downstream DNA
linker
Thumb
NTE0
N-ZnF
RT
Myb
upstream DNA
DNA top strand
DNA bottom strand
Eubacterium rectale group IIC intron
PDB 7UIN
splicing active site
intron RNA
DNA binding
domain
Thumb
NTE0
DNA hairpin target
RT
RNA
domain IV
B
Bombyx mori R2 retrotransposon
RLE endonuclease
CCHC
ZnF
NTE0
Thumb
RT
Myb
upstream DNA
DNA top strand
DNA bottom strand
Homo sapiens LINE-1 ORF2 (AlphaFold2 model)
endonuclease active site
Cys-rich
doman
CCHC
ZnF
APE endonuclease
APE+DNA
crystal structure
PDB 7N8S
modelled
from R2Bm
structure
DNA modelled
from R2Bm
structure
Thumb
RT
120°
endonuclease active site
downstream DNA
CCHC
ZnF
NTE0
linker
120°
CCHC
ZnF
NTE0
linker
DNA modelled
from R2Bm
structure

Fig. S5. Comparisons between the R2Bm TPRT complex and related structures. (**A**) Comparison to the group IIC intron structure (*19*). The group IIC intron DNA hairpin is coloured by its alignment to the bottom and top strands of the R2Bm target. (**B**) Comparison to a model of the human LINE-1 ORF2. The model was created by superimposing an AlphaFold model of ORF2 (AlphaFold database O00370) with the crystal structure of the LINE-1 ORF2 APE domain in complex with target DNA (*35*), and then adding part of the target DNA from the R2Bm structure.

Fig. S6

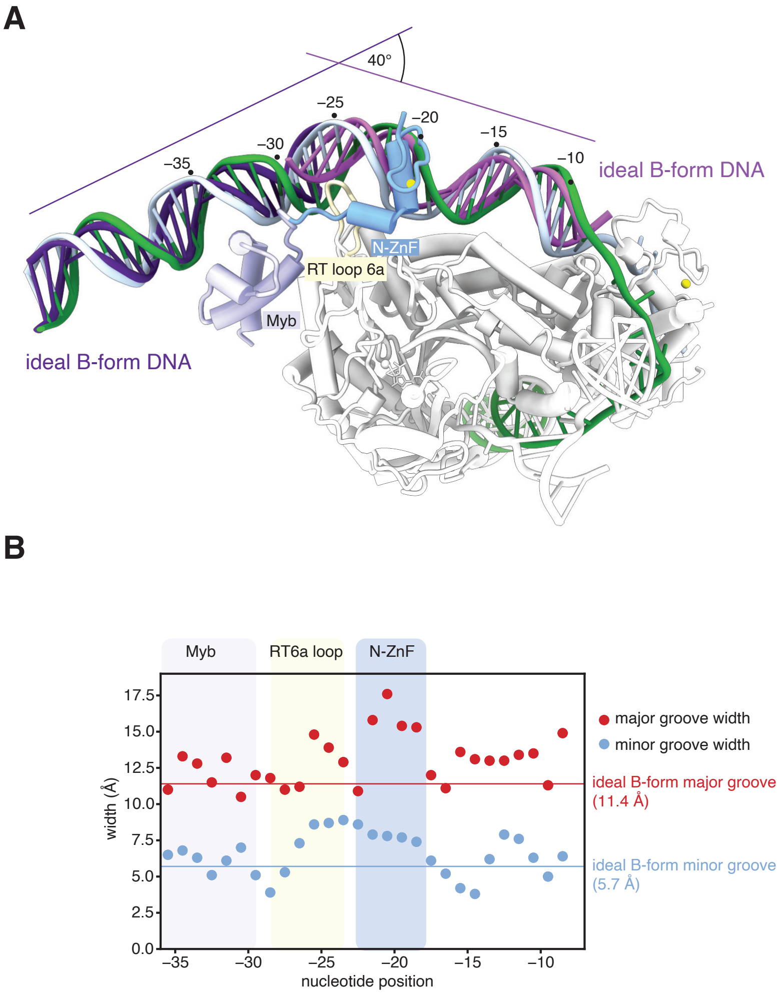

Fig. S6. Upstream target distortion by R2Bm. (**A**) Overlay of the R2Bm structure with two idealized B-form DNA helices. (**B**) Major and minor groove widths calculated using 3DNA *(45)*. Both grooves are widened at the N-ZnF binding site and at the point of bending.

Fig. S7

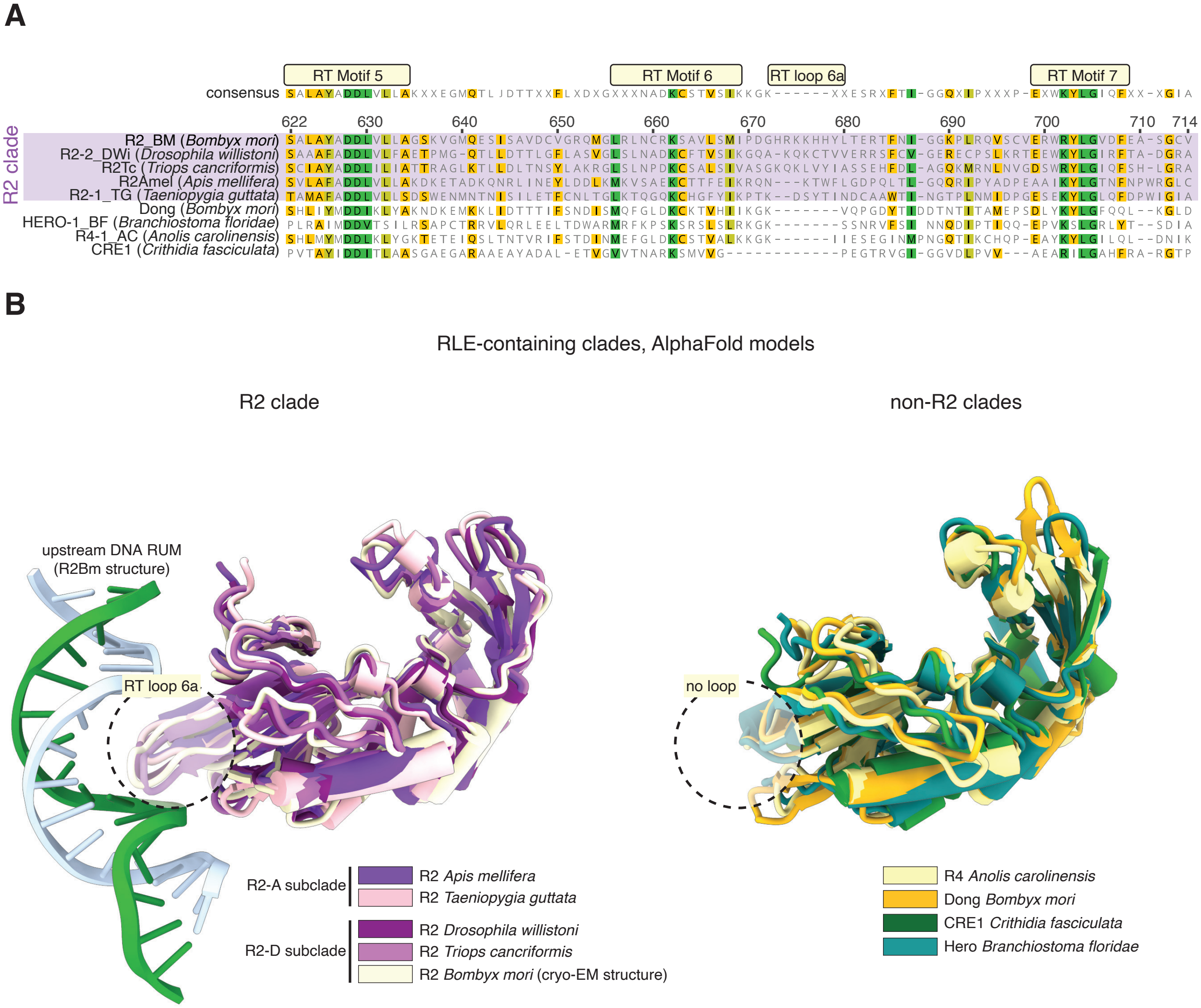

Fig. S7. Comparison of RLE-clade non-LTR retrotransposon reverse transcriptase domains. (**A**) Multiple sequence alignment of motifs 5 – 7. In addition to R2Bm, two representatives of the R2-D clade (R2-2_DWi and R2Tc) and two representatives of the R2-A clade (R2AmeI, R2-1_TG) were chosen, along with four representatives of non-R2 RLE-clade RTs. Sequences were aligned with MAFFT. (**B**) AlphaFold models for the representative sequences, superimposed on the cryo-EM structure of R2Bm RT. All R2-clade RTs investigated had a 6a loop, while no non-R2 RTs had this loop.

Fig. S8

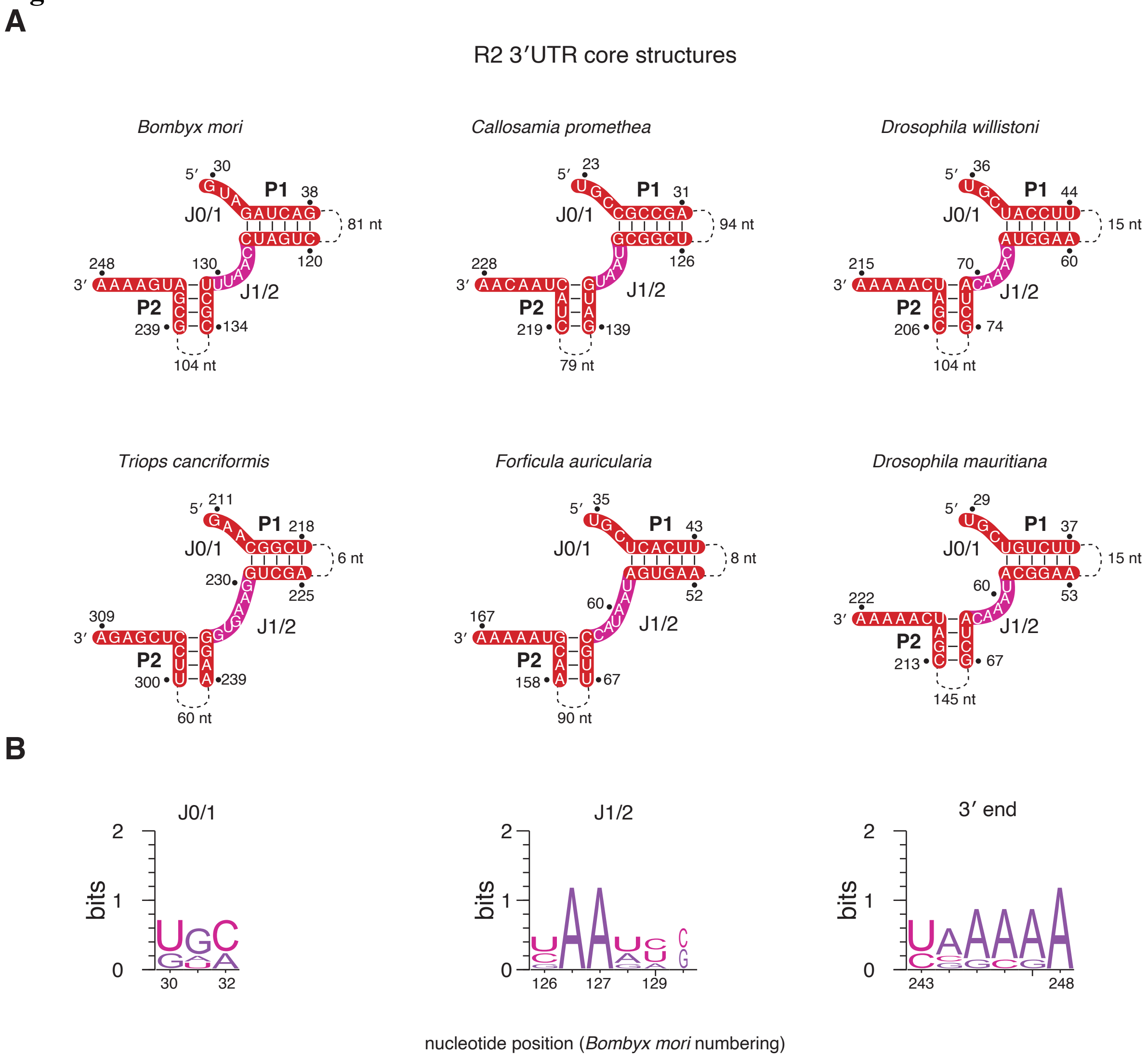

Fig. S8. Comparison of R2 3′ UTR secondary structures. (**A**) Core secondary structures, corresponding to the bases visible in our cryo-EM map. Secondary structures are adapted from (26), except for *Triops cancriformis* which was calculated from covariance analysis with R2La (*Lepidurus arcticus*), R2LcB (*Lepidurus couesii*) and R2Ll (*Lepidurus apus lubbocki*). (**B**) Sequence logos for the single stranded regions of these six RNAs.

Fig. S9

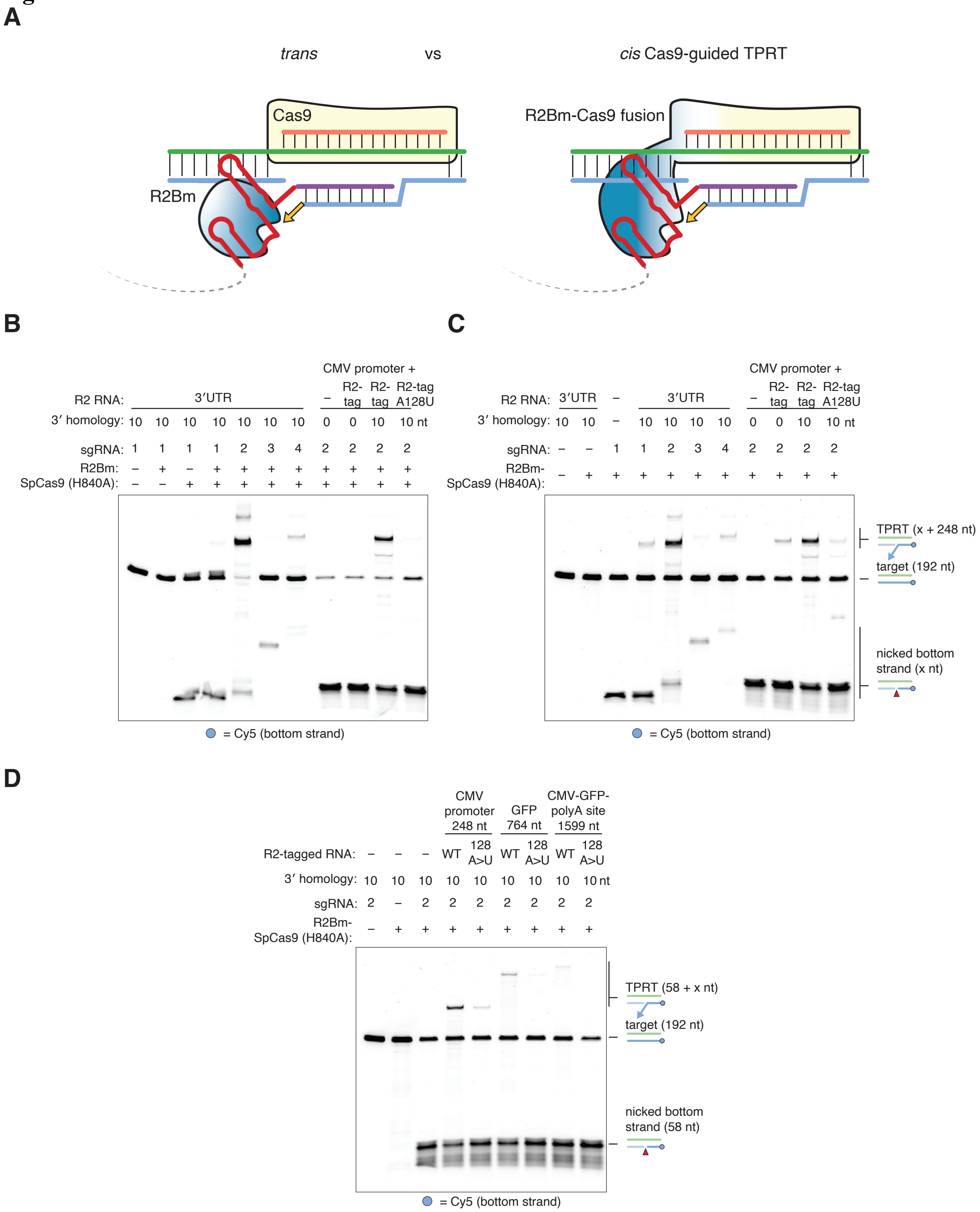

Fig. S9. Related to Fig. 5. Comparison of *trans* vs *cis* Cas9-directed TPRT. (**A**) Schematic of *trans* vs *cis* Cas9 TPRT. (**B**) Denaturing gel of in vitro TPRT reactions on the labeled 192 bp *Drosophila virilis* target (see Fig. 5). SpCas9 and R2Bm are supplied in *trans*. sgRNAs are

numbered as in Fig. 5; all R2 RNAs or R2-tagged RNAs have 10 nt of 3′ homology to the nick site of the sgRNA. The gel was visualized by Cy5 fluorescence. (**C**) the same as (B) but with the R2Bm-SpCas9 fusion. (**D**) The R2Bm-Cas9 fusion can perform TPRT at the *D. virilis* target of gene-sized insertions,

Fig. S10

A

Bombyx mori genome scan

chromosome	accession	length (Mb)	profile matches	exact matches
1	CP114807.1	20.7	4253	0
2	CP114808.1	8.4	1305	0
3	CP114809.1	15.2	2771	0
4	CP114810.1	18.9	3767	0
5	CP114811.1	19.0	3775	0
6	CP114812.1	16.8	3188	0
7	CP114813.1	14.0	2563	0
8	CP114814.1	16.3	3042	0
9	CP114815.1	17.0	3237	0
10	CP114816.1	17.7	3524	0
11	CP114817.1	20.5	4014	10 → all in 28S rDNA array
12	CP114818.1	17.8	3249	0
13	CP114819.1	17.9	3433	0
14	CP114820.1	13.5	2470	0
15	CP114821.1	18.5	3522	0
16	CP114822.1	14.4	2528	0
17	CP114823.1	16.9	3121	0
18	CP114824.1	15.8	2979	0
19	CP114825.1	14.9	2737	0
20	CP114826.1	12.5	2180	0
21	CP114827.1	15.4	2897	0
22	CP114828.1	18.5	3547	0
23	CP114829.1	21.5	4067	0
24	CP114830.1	18.0	2912	0
25	CP114831.1	14.6	2717	0
26	CP114832.1	11.6	2083	0
27	CP114833.1	10.9	1923	0
28	CP114834.1	10.6	1788	0
w	CP114835.1	8.6	775	0

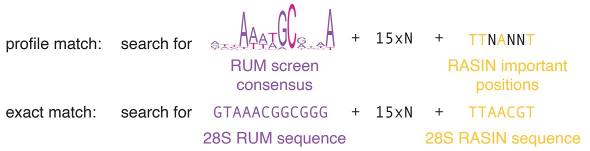

B

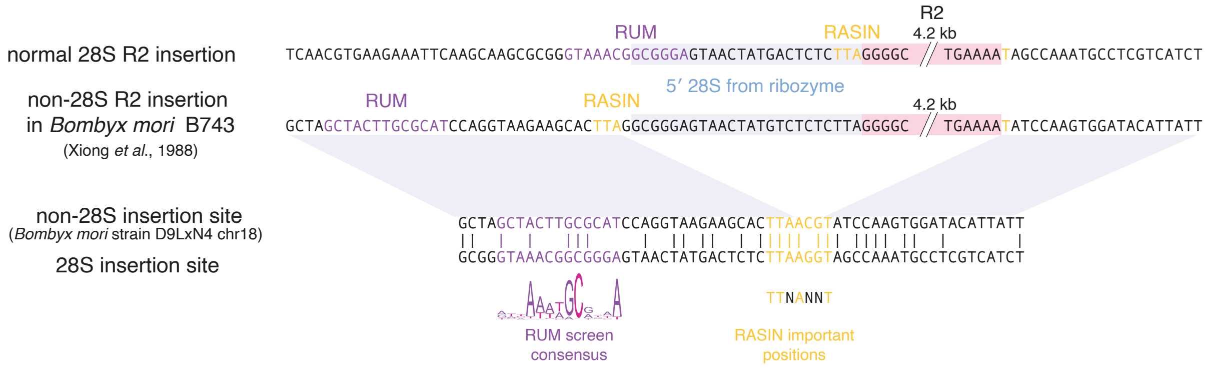

C

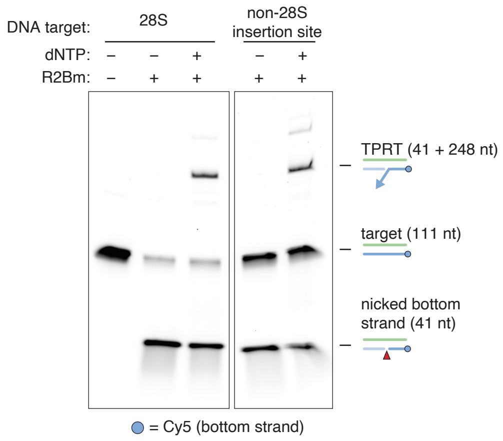

Fig. S10. Insertion of R2Bm outside of its 28S target site. (**A**) Potential off-target insertion sites in the *Bombyx mori* genome. Profile matches: the genome was scanned with FIMO (*46*), using a profile derived from the RUM screen, a 15N spacer, and the important RASIN positions identified in Fig. 3D. All matches with $p < 0.00001$ (FDR = 0.484) were counted. Exact matches: matches to the precise RUM and RASIN sequence found in the 28S ribosomal DNA. (**B**) Comparison of a typical insertion of R2Bm at a 28S locus to the insertion noted by Eickbush and colleagues in *Bombyx mori* strain B743 (*30*). The uninserted site could be identified by BLAST searching of sequenced *Bombyx mori* genomes. The non-28S insertion also inserts 24 nt of 28S sequence at the target (blue highlighting), which derives from the 5' 28S homology present on the R2Bm RNA after ribozyme cleavage from the nascent rRNA (*47*). (**C**) This uninserted site shows lower but still substantial TPRT activity compared to the 28S target.

Table S1. Cryo-EM data collection, refinement, and validation statistics

	R2Bm TPRT complex<br>(PDB 8GH6)<br>(EMDB 40033)<br>(EMPIAR-11458)
**Data collection and Processing**	
Microscope	Thermo Scientific Titan Krios G3i cryo-TEM
Voltage (keV)	300
Camera	Gatan K3
Magnification	130,000x
Pixel size at detector (Å/pixel)	0.663
Total electron exposure (e^{-}/Å^2)	42.2
Exposure rate (e-/pixel/sec)	26.9
Number of frames collected during exposure	40
Defocus range (μm)	−2.5 to −1.5
Automation software	EPU
Energy filter slit width	20 eV
Micrographs collected (no.)	16,551
Total extracted particles (no.)	1,716,620
**3D reconstruction:**	
Refined particles (no.)	39,616
Estimated error of translations/rotations	0.44 Å / 1.07°
Resolution (global, Å)	
FSC 0.5 (unmasked/masked)	7.58 / 3.63
FSC 0.143 (unmasked/masked)	3.90 / 3.08
Resolution range (local, Å)	3.10 – 5.89 (5th to 95th percentiles within refinement mask)
Map sharpening *B* factor (Å^2)	−47.9
**Model composition**	
Non-hydrogen atoms	10785
Protein residues	898
Ligands	4
RNA/DNA bases	177
**Model Refinement**	
Refinement package	phenix.real_space_refine
- resolution cutoff	3 Å
Model-Map scores	
-CC	0.81
-Model-Map FSC = 0.5	3.2 Å
Mean *B* factors (Å^2)	
Protein residues	43.1
Ligands	35.2
RNA/DNA	221.0
R.m.s. deviations from ideal values	
Bond lengths (Å)	0.006
Bond angles (°)	1.095
**Validation**	
MolProbity score	0.86
CaBLAM outliers (%)	1.13
Clashscore	1.15
Rotamer outliers (%)	0.13
C-beta outliers (%)	0
EMRinger score	3.69
Ramachandran plot	

Favored (%)	97.9
Outliers (%)	0.11
Nucleic acid geometry	
Correct sugar puckers (%)	97.4
Good backbone conformations (%)	79.5

Table S2. Plasmid maps for experiments in this study.

Plasmid name	Link to plasmid map in Benchling
pET-His-MBP-SUMO-R2Bm-SII	https://benchling.com/s/seq-mDQasLPGqprvY2q3Z3RX?m=slm-WwE4YIfqJIHdi9RkXvMM300
pET-His-MBP-SUMO-R2Bm-SII deltaN mutant	https://benchling.com/s/seq-7QfOOvPXrdjXDz5O5scl?m=slm-BhN2OvHrzcfoPgQOcQ1w
pET-His-MBP-SUMO-R2Bm-SII delRT6a mutant	https://benchling.com/s/seq-dDNXmMf22LpU830q0GH2?m=slm-z8w8VvaPkMjKBODhcXXU
pET-His-MBP-SUMO-R2Bm-SII R902A D903A mutant	https://benchling.com/s/seq-3x3DMd5EdzKseIslcwUX?m=slm-CmSKlDgCg0dv4PllQ70x
pET-His-MBP-SUMO-R2Bm-33XTEN-SpCas9(H840A)-SII	https://benchling.com/s/seq-Rg9wDeGwkCX95YJqbHKH?m=slm-By2wtNUI6zeDgdPmUro1
pACYC-28S rDNA	https://benchling.com/s/seq-41DWKQbG9QQ9WRKgg26A?m=slm-3uBk4kIxXPyEzKxDkrSq
pACYC-28S rDNA no-RASIN mutant	https://benchling.com/s/seq-Y999Ik3Oh0r4kgnfQmDg?m=slm-Zgl6HTMV5FS4FhiShLDZ
Drosophila virilis target	https://benchling.com/s/seq-zRz7TrHTTealAkv8QTPr?m=slm-YpP2qfgaJheFwnGkgyz5
pRS426-R2Bm RNA	https://benchling.com/s/seq-z5ujo80r011VextOQbvs?m=slm-AmjKZhNsEsZXTh4oS7XC
CMVP-R2tag	https://benchling.com/s/seq-9rZmlFLH8dibwDsqwX8O?m=slm-7CSl5F4Fqnh0PWZrg0I7
GFP-R2tag	https://benchling.com/s/seq-RcEhJa2QbZxLtPjZOnBe?m=slm-QXYFl2gx7Pb5xlBMJY5r
CMVP-GFP-polyA-R2tag	https://benchling.com/s/seq-mQU0Qfsnmi6KbNtNToZO?m=slm-zKnJTCmpsiPjNZvgVuNH

Table S3. RNA sequences for experiments in this study.

RNA name	Sequence	1st Fig.
R2 3′UTR + **up to 15 nt 28S 3′ homology**	GCCUUGCACAGUAGUCCAGCGGUAAGGGUGUAGAUCAGGCCCGUCUGUUUCUCCCCCGG<br>AGCUCGCUCCCUUGGCUUCCCUUAUAUAUUUUAACAUCAGAAACAGACAUUAAACAUCU<br>ACUGAUCCAAUUUCGCCGGCGUACGGCCACGAUCGGGAGGGUGGGAAUCUCGGGGGUCU<br>UCCGAUCCUAAUCCAUGAUGAUUACGACCUGAGUCACUAAAGACGAUGGCAUGAUGAUC<br>CGGCGAUGAAAA**UAGCCAAAUGCCUCG**	1C
R2 tag	GGUAGAUCAGUUCGCUGAUCCAAUUUCGCUUCGGCGAUGAAAA	4E
CMV promoter + **R2 tag**	GGUGAUGCGGUUUUGGCAGUACAUCAAUGGGCGUGGAUAGCGGUUUGACUCACGGGGAU<br>UUCCAAGUCUCCACCCCAUUGACGUCAAUGGGAGUUUGUUUUGGCACCAAAAUCAACGG<br>GACUUUCCAAAAUGUCGUAACAACUCCGCCCCAUUGACGCAAAUGGGCGGUAGGCGUGU<br>ACGGUGGGAGGUCUAUAUAAGCAGAGCUCUCUGGCUAACUAGAGAACCCACUGCUUACU<br>GGC**GGUAGAUCAGUUCGCUGAUCCAAUUUCGCUUCGGCGAUGAAAA**	4G
GFP + **R2 tag**	GAUGGUGAGCAAGGGCGAGGAGCUGUUCACCGGGGUGGUGCCCAUCCUGGUCGAGCUGG<br>ACGGCGACGUAAACGGCCACAAGUUCAGCGUGUCCGGCGAGGGCGAGGGCGAUGCCACC<br>UACGGCAAGCUGACCCUGAAGUUCAUCUGCACCACCGGCAAGCUGCCCGUGCCCUGGCC<br>CACCCUCGUGACCACCCUGACCUACGGCGUGCAGUGCUUCAGCCGCUACCCCGACCACA<br>UGAAGCAGCACGACUUCUUCAAGUCCGCCAUGCCCGAAGGCUACGUCCAGGAGCGCACC<br>AUCUUCUUCAAGGACGACGGCAACUACAAGACCCGCGCCGAGGUGAAGUUCGAGGGCGA<br>CACCCUGGUGAACCGCAUCGAGCUGAAGGGCAUCGACUUCAAGGAGGACGGCAACAUCC<br>UGGGGCACAAGCUGGAGUACAACUACAACAGCCACAACGUCUAUAUCAUGGCCGACAAG<br>CAGAAGAACGGCAUCAAGGUGAACUUCAAGAUCCGCCACAACAUCGAGGACGGCAGCGU<br>GCAGCUCGCCGACCACUACCAGCAGAACACCCCCAUCGGCGACGGCCCCGUGCUGCUGC<br>CCGACAACCACUACCUGAGCACCCAGUCCGCCCUGAGCAAAGACCCCAACGAGAAGCGC<br>GAUCACAUGGUCCUGCUGGAGUUCGUGACCGCCGCCGGGAUCACUCUCGGCAUGGACGA<br>GCUGUACAAGUAA**GGUAGAUCAGUUCGCUGAUCCAAUUUCGCUUCGGCGAUGAAAA**	4G
R2 3′UTR + **5 nt no-RASIN target homology**	GCCUUGCACAGUAGUCCAGCGGUAAGGGUGUAGAUCAGGCCCGUCUGUUUCUCCCCCGG<br>AGCUCGCUCCCUUGGCUUCCCUUAUAUAUUUUAACAUCAGAAACAGACAUUAAACAUCU<br>ACUGAUCCAAUUUCGCCGGCGUACGGCCACGAUCGGGAGGGUGGGAAUCUCGGGGGUCU<br>UCCGAUCCUAAUCCAUGAUGAUUACGACCUGAGUCACUAAAGACGAUGGCAUGAUGAUC<br>CGGCGAUGAAAA**AAUCA**	5C
no-RASIN sgRNA (**spacer highlighted**)	G**GGCAUUUGGCGUUGAUUAGG**GUUUUAGAGCUAGAAAUAGCAAGUUAAAAUAAGGCUAG<br>UCCGUUAUCAACUUGAAAAAGUGGCACCGAGUCGGUGCUUUUUU	5C
D. virilis sgRNA 1	G**CUUCCGUUGAUAUUUAUUGA**GUUUUAGAGCUAGAAAUAGCAAGUUAAAAUAAGGCUAG<br>UCCGUUAUCAACUUGAAAAAGUGGCACCGAGUCGGUGCUUUUUU	5D
D. virilis sgRNA 2	G**UUGAUAUUUAUUGAUGGCUC**GUUUUAGAGCUAGAAAUAGCAAGUUAAAAUAAGGCUAG<br>UCCGUUAUCAACUUGAAAAAGUGGCACCGAGUCGGUGCUUUUUU	5D
D. virilis sgRNA 3	G**AUAUUUCAAUAUUUAUUUGU**GUUUUAGAGCUAGAAAUAGCAAGUUAAAAUAAGGCUAG<br>UCCGUUAUCAACUUGAAAAAGUGGCACCGAGUCGGUGCUUUUUU	5D
D. virilis sgRNA 4	G**UUUAUUUGUUGGCUGGCUCG**GUUUUAGAGCUAGAAAUAGCAAGUUAAAAUAAGGCUAG<br>UCCGUUAUCAACUUGAAAAAGUGGCACCGAGUCGGUGCUUUUUU	5D
R2 3′UTR + **10 nt *D. virilis* sgRNA 1 nick homology**	GCCUUGCACAGUAGUCCAGCGGUAAGGGUGUAGAUCAGGCCCGUCUGUUUCUCCCCCGG<br>AGCUCGCUCCCUUGGCUUCCCUUAUAUAUUUUAACAUCAGAAACAGACAUUAAACAUCU<br>ACUGAUCCAAUUUCGCCGGCGUACGGCCACGAUCGGGAGGGUGGGAAUCUCGGGGGUCU<br>UCCGAUCCUAAUCCAUGAUGAUUACGACCUGAGUCACUAAAGACGAUGGCAUGAUGAUC<br>CGGCGAUGAAAA**AUAAAUAUCA**	5E
R2 3′UTR + **10 nt *D. virilis* sgRNA 2 nick homology**	GCCUUGCACAGUAGUCCAGCGGUAAGGGUGUAGAUCAGGCCCGUCUGUUUCUCCCCCGG<br>AGCUCGCUCCCUUGGCUUCCCUUAUAUAUUUUAACAUCAGAAACAGACAUUAAACAUCU<br>ACUGAUCCAAUUUCGCCGGCGUACGGCCACGAUCGGGAGGGUGGGAAUCUCGGGGGUCU<br>UCCGAUCCUAAUCCAUGAUGAUUACGACCUGAGUCACUAAAGACGAUGGCAUGAUGAUC<br>CGGCGAUGAAAA**CCAUCAAUAA**	5E
R2 3′UTR + **10 nt *D. virilis* sgRNA 3 nick homology**	GCCUUGCACAGUAGUCCAGCGGUAAGGGUGUAGAUCAGGCCCGUCUGUUUCUCCCCCGG<br>AGCUCGCUCCCUUGGCUUCCCUUAUAUAUUUUAACAUCAGAAACAGACAUUAAACAUCU<br>ACUGAUCCAAUUUCGCCGGCGUACGGCCACGAUCGGGAGGGUGGGAAUCUCGGGGGUCU<br>UCCGAUCCUAAUCCAUGAUGAUUACGACCUGAGUCACUAAAGACGAUGGCAUGAUGAUC<br>CGGCGAUGAAAA**AAUAAAUAUU**	5E

R2 3′UTR + **10 nt *D. virilis* sgRNA 4 nick homology**	GCCUUGCACAGUAGUCCAGCGGUAAGGGUGUAGAUCAGGCCCGUCUGUUUCUCCCCCGG<br>AGCUCGCUCCCUUGGCUUCCCUUAUAUAUUUUAACAUCAGAAACAGACAUUAAACAUCU<br>ACUGAUCCAAUUUCGCCGGCGUACGGCCACGAUCGGGAGGGUGGGAAUCUCGGGGGUCU<br>UCCGAUCCUAAUCCAUGAUGAUUACGACCUGAGUCACUAAAGACGAUGGCAUGAUGAUC<br>CGGCGAUGAAAA**GCCAGCCAAC**	5E
CMV promoter + **R2 tag** + 10 nt *D. virilis* sgRNA 2 nick homology	GGUGAUGCGGUUUUGGCAGUACAUCAAUGGGCGUGGAUAGCGGUUUGACUCACGGGGAU<br>UUCCAAGUCUCCACCCCAUUGACGUCAAUGGGAGUUUGUUUUGGCACCAAAAUCAACGG<br>GACUUUCCAAAAUGUCGUAACAACUCCGCCCCAUUGACGCAAAUGGGCGGUAGGCGUGU<br>ACGGUGGGAGGUCUAUAUAAGCAGAGCUCUCUGGCUAACUAGAGAACCCACUGCUUACU<br>GGC**GGUAGAUCAGUUCGCUGAUCCAAUUUCGCUUCGGCGAUGAAAA**CCAUCAAUAA	5E
CMV promoter + **R2 tag** + 10 nt *D. virilis* sgRNA 2 nick homology, A128U	GGUGAUGCGGUUUUGGCAGUACAUCAAUGGGCGUGGAUAGCGGUUUGACUCACGGGGAU<br>UUCCAAGUCUCCACCCCAUUGACGUCAAUGGGAGUUUGUUUUGGCACCAAAAUCAACGG<br>GACUUUCCAAAAUGUCGUAACAACUCCGCCCCAUUGACGCAAAUGGGCGGUAGGCGUGU<br>ACGGUGGGAGGUCUAUAUAAGCAGAGCUCUCUGGCUAACUAGAGAACCCACUGCUUACU<br>GGC**GGUAGAUCAGUUCGCUGAUCCAUUUUCGCUUCGGCGAUGAAAA**CCAUCAAUAA	5E
